# Policy Responses to the COVID‐19 Pandemic in High‐Income Countries and the Associated Maternal‐Infant Health Outcomes: A Systematic Literature and Policy Review

**DOI:** 10.1002/hsr2.71523

**Published:** 2026-05-15

**Authors:** Ashleigh Shipton, Katie Mcbain, Melissa Wake, Sharon Goldfeld, Fiona Mensah

**Affiliations:** ^1^ Department of Paediatrics University of Melbourne Melbourne Australia; ^2^ Generation Victoria, Murdoch Children's Research Institute Melbourne Australia; ^3^ Centre for Community Child Health, Royal Children's Hospital Melbourne Australia; ^4^ Population Health, Murdoch Children's Research, Institute Melbourne Australia; ^5^ Intergenerational Health Group, Murdoch Children's Research Institute Melbourne Australia; ^6^ Women and Kids, South Australian Health and Medical Research Institute Adelaide Australia

**Keywords:** COVID‐19, infant health, maternal health, Oxford COVID‐19 Government Response Tracker Stringency Index, policy, systematic review

## Abstract

**Background:**

Foreseeing how policy impacts pregnant women and infants is limited by ethical challenges of experimental research within these groups. The COVID‐19 pandemic generated natural experiments, offering rare opportunities to explore associations between specific policy responses and maternal‐infant health outcomes. These insights are useful for informing policy design aligned with the 2030 Sustainable Development Goals.

**Aims:**

To systematically review evidence of associations between COVID‐19 policies, OxCGRT Stringency Index (a University of Oxford tool for comparing the stringency of policies) and maternal‐infant health outcomes during pregnancy, birth, and the first 12 months postpartum from 2020‐2022 in high‐income countries.

**Methods:**

Following PRISMA guidelines, Embase, MEDLINE, PubMed, and Web of Science were searched (January 1 2020‐July 9 2022) using subject headings, keywords, and variants for “COVID‐19”, “policies”, “perinatal”, and “randomized”, and “non‐randomized” study designs. Eligible studies were from high‐income countries, published in English, and used comparison groups. Two reviewers independently extracted and analyzed data. Heterogeneity and risk of bias made meta‐analysis inappropriate. Outcomes were instead reported using tables and visuals of effect sizes, ratio estimates, and 95% confidence intervals.

**Results:**

Of 3143 citations, 35 studies met inclusion criteria. All reported high OxCGRT Stringency Index during exposure, validating the fidelity of adhering to defined policy exposure periods. Lockdown was associated with reductions in preterm and low birthweight births, infant emergency admissions, and breast feeding women, and increases in hypertensive disorders of pregnancy and gestational diabetes mellitus. Telehealth was associated with earlier gestational age at antenatal visits and less breast feeding women. Maternal mental health and stillbirth results were mixed.

**Conclusion:**

High stringency policies, including lockdowns and telehealth, were associated with both favorable and adverse maternal‐infant outcomes. Findings inform avenues for future causal inference research and areas for mitigation planning should high stringency policies be reintroduced, supporting progress towards the 2030 Sustainable Development Goals.

## Summary

- Study question
  ∘ To what extent were maternal‐infant outcomes associated with policies responding to the COVID‐19 pandemic in high‐income countries?
- What's already known
  ∘ Previous reviews have examined maternal and infant outcomes during the COVID‐19 pandemic, but most have treated the pandemic period as a single undifferentiated exposure or focused on only one lifecourse stage within the perinatal period. Few have isolated the associations of specific policy interventions across both antenatal and postnatal periods.
- What this study adds
  ∘ By applying strict exposure definitions and using the OxCGRT Stringency Index, this review synthesizes associations between discrete COVID‐19 policies (e.g., lockdown, telehealth) and perinatal outcomes across the lifecourse. Lockdowns were associated with decreases in preterm births, infant emergency admissions and breast feeding, and increases in hypertensive disorders of pregnancy and gestational diabetes mellitus. Telehealth was associated with a younger age at initial antenatal visits and a lower number of breast feeding women.

## 1 Background

Pregnant women and infants are particularly vulnerable to health and economic crises [1, 2]. Their health outcomes are highly susceptible to social, economic and political shifts [3]. None have been more seismic globally since World War II than those following the COVID‐19 pandemic declared March 11, 2020 [4]. This triggered rapid policy responses by governments and healthcare services worldwide with the intention to slow transmission of SARS‐CoV‐2, stabilize economies and avoid overwhelming health services. These policies fell under three categories: containment and closure (stay‐at‐home requirements or “lockdown,” border closures, work, school and childcare closures); economic (welfare subsidies, medical insurance extensions and offsets in rent and mortgage repayments); and public health measures (shortened postnatal hospital stays, visitor restrictions to appointments, telehealth, and personal protective equipment) [5].

Evidence underpinning policies and services for pregnant women and infants is limited by the ethical challenges of subjecting this cohort to experimental design. The onset of the pandemic and policies created an extraordinary period of transformation in the conditions that influence health, providing natural experiments that offer a unique opportunity to investigate causal drivers of maternal‐infant health outcomes. Findings could support future pandemic preparedness and more generally progress the 2030 Sustainable Development Goals (SDGs) [6].

Several systematic reviews and meta analyses have examined maternal and infant health during the COVID‐19 pandemic, often focusing on biological exposures such as SARS‐CoV‐2 infection or vaccination or assessing outcomes across the general pandemic period without isolating specific policies [7, 8, 9, 10, 11, 12, 13, 14, 15, 16, 17]. However, none have examined the associations between specific policy responses (that represent structural changes influencing the social determinants of health) and a broad range of maternal and infant outcomes spanning the whole perinatal period. The primary aim of this systematic review was to synthesize the collective quantitative evidence available to July 9, 2022 on the extent to which a range of specific COVID‐19 policies were associated with health outcomes during the perinatal period (pregnancy, birth, and the first 12 months postpartum) for women and their infants in high‐income countries (HIC's). The secondary aim was to identify the Oxford COVID‐19 Government Response Tracker (OxCGRT) Stringency Indices for each included study to validate our methodology of aligning natural experiment analyses as closely as possible with the principles of the gold standard randomized control trials (RCT's).

## 2 Methods

A systematic and comprehensive search strategy was completed according to the PRISMA guidelines [18]. A protocol was developed a priori and registered with PROSPERO (CRD42022343049). Ethics approval was received from the Royal Children's Hospital Human Research Ethics Committee (87751) as part of a broader project.

### 2.1 Study Selection

MEDLINE, Embase, Web of Science and PubMed databases were searched from January 1, 2020, to July 9, 2022. The search included relevant MeSH and subject heading terms, keywords and word variants for “COVID‐19”, “policies”, “perinatal”, and “randomized” and “non‐randomized” study designs (Supporting Information S1: Appendix SA). Boolean operators were used within concept groups (using “OR”) and between concept groups (using “AND”).

Eligibility criteria were based on the population, exposure, comparison, and outcome methodology recommended by Cochrane (Table 1) [19]. Our search criteria were inclusive of randomized study designs and non‐randomized study designs with comparison groups, such as cohort studies, case‐control studies, epidemiological studies, interrupted‐time series, and natural experiments, although the final evidence review did not include any applicable randomized study designs.

**Table 1:** Study eligibility criteria.

	Inclusion criteria	Exclusion criteria
Population	General population of pregnant women, or mothers 0‐12 months postpartum, or infants 0‐12 months of age living in a high‐income country at the time of the COVID‐19 pandemic according to The World Bank classification [20].	Condition‐specific populations; pregnant women, mothers and infants living in low‐ or middle‐income countries at the time of the COVID‐19 pandemic; pooled age group data so that infants 0‐12 months of age are included within wider paediatric age ranges.
Exposure	Had to be systemically exposed to a specific COVID‐19 policy.	Focus is on exposure to the SARS‐CoV‐2 virus, the COVID‐19 vaccinations or the general COVID‐19 pandemic period rather than a specific COVID‐19 policy period. To reduce the risk of non‐exposure in the “exposed” group, studies were excluded in instances where the study period for the “exposed” group went beyond 14 days before or after the duration that the COVID‐19 policy was in effect.
Comparison	Had to not be exposed to this policy during a comparable life stage that could be during or before the pandemic.	No comparison group.
Outcome	Health outcomes during pregnancy, birth, or the first 12 months postnatally. The term “health” in this review is broad; for example, studies could include mental health, physical health, nutritional health, developmental health and indirect outcomes that determine health such as access and use of health services.	

We limited this review to HIC's, according to The World Bank classification [20], because of the disparities across low‐, middle‐, and HICs regarding perinatal health service provision, capacity to enforce COVID‐19 policies and access to COVID‐19 vaccinations which could directly introduce further confounding to already non‐randomized studies [7, 8, 10]. We limited studies to the English language due to resource constraints.

Observational studies, such as natural experiments, often lack clear exposure timing making causal inference challenging. For this review, we ensured a clear distinction between the exposed and comparison groups by explicitly defining strict criteria for exposure periods to as closely as possible emulate the methodology of the gold standard for causal inference ‐ an RCT. Participants in the “exposed” group were those who had been systemically exposed to a specific COVID‐19 policy; the control group were not exposed to this policy during a comparable life stage that could be during or before the pandemic. To reduce the risk of non‐exposure in the “exposed” group, studies were excluded in instances where the study period for the “exposed” group went beyond 14 days before or after the duration that the COVID‐19 policy was in effect.

### 2.2 Oxford COVID‐19 Government Response Tracker Stringency Index

We extracted data on how policy varied across countries. For containment and closure policy, we derived a minimum and maximum OxCGRT Stringency Index [21, 22]. This index is a cross‐country and cross‐temporal measure developed by the University of Oxford to numerically quantify the stringency of government containment and closure policies from 0 (least stringent) to 100 (most stringent). Author AS was a member of the coding team who developed this index and the codebook and equations they used are publicly available [22]. For telehealth policies, we recorded what format the telehealth entailed (i.e., phone and/or video) and if there was any home monitoring provided (i.e., blood pressure machines).

### 2.3 Article Screening

Two researchers (AS, KM) with expertise in paediatric medicine and psychology, respectively, completed the screening of all titles, abstracts and potentially relevant full texts using Covidence™ software [23]. This process was conducted independently and tested in a pilot phase before implementation. Conflicts were resolved by consensus with reference to the eligibility criteria or through consultation with another researcher with expertise in biostatistics and epidemiology (FM).

### 2.4 Data Extraction

Two researchers (AS, KM) independently extracted data from five purposively selected studies using Covidence™ software to pilot the process [23]. Conflicts were resolved by consensus with reference to the study protocol or with a third researcher (FM). Once concordance was achieved, AM and FM continued an extensive process of data extraction and analysis independently using a predefined template (Supporting Information S1: Appendix SB). Subgroup analyses were not extracted unless full population results were unavailable. FM and AS identified exclusions according to: (i) no clear rationale for being a health outcome (e.g., duration of labor); (ii) not a meaningful health outcome for practice (e.g., mean birthweight, unlike low birthweight which has risks associated and was included): (iii) only one estimate for an exposure‐outcome combination; (iv) quality of care rather than health outcomes (e.g., postnatal duration of stay in hospital). If a study had comparison groups from both before and during the pandemic (such as a period during the pandemic but with no lockdown), the former was preferenced in the synthesis. Pre‐pandemic comparison groups were prioritized to minimize exposure to unmeasured pandemic policies, such as ongoing social restrictions which may have continued during non‐lockdown periods.

As recommended by Cochrane and STROBE, detailed numerical health outcome data was extracted [19] including; absolute numbers of participants and events, summary estimates and measures of precision ‐ 95% confidence intervals (CI), variance, or standard deviation; between‐group estimates with measures of precision; and key conclusions [19, 24]. Adjusted estimates were the preference where available [19].

If health outcome data required derivation to a more comparable form, statistical calculations were made using Stata™ software [25]. Odds ratios were derived from study proportions to be comparable with most studies reporting adjusted or unadjusted odds ratios. If a reference group was not comparable to other studies, estimates were re‐derived, e.g., estimates from a study that reported on very preterm births with a reference group to all preterm births, were rederived with a reference group of all births. This process was completed independently by two researchers to reach consensus (AS, FM). If there was insufficient data for extraction or derivation of estimates, then an attempt was made to contact the authors of the study to obtain this data.

### 2.5 Statistical Analysis

In accordance with Cochrane guidelines, meta‐analysis was not appropriate due to the methodological diversity and concerns of bias [19]. Instead, tables and visual displays provide structured reporting of health outcomes [19]. Estimated mean differences and their 95% CI were transformed to effect size estimates to enable comparability across measures with varying scales. Similarly, odds ratio or other ratio estimates were log‐transformed to establish a common null value of zero within the visual plots.

Results were reported following Greenland et al. (2016) [26]. “Association” was used over “effect” to emphasize limitations in inferring causality with non‐randomized studies. The aim of reporting was to provide an evaluation of “certainty” rather than “significance” so estimates and CIs were used rather than *p* values.

### 2.6 Quality Assessment

Risk of bias was assessed using the Newcastle‐Ottawa Scale (NOS) endorsed by Cochrane [27]. Two researchers (AS, KM) independently evaluated five diverse studies, resolving conflicts by consensus with reference to the NOS criteria or consultation with a third researcher (FM). Once concordance was reached, AS completed assessments in consultation with FM. Studies were classified as “high”, “moderate”, or “low” risk of bias (details in Supporting Information S1: Appendix SC).

Accounting for key confounders is essential in the design and analysis of non‐randomized studies, whereby a “target trial” design may be emulated by addressing important predisposing factors typically balanced by randomization [28]. Hartling et al. (2012) [29] emphasizes the importance of transparency in identifying which confounders are important for each systematic review. In this systematic review, maternal age stood out as the most important confounder due to well‐established associations with adverse perinatal outcomes, education, and social capital [30, 31, 32, 33]. Further relevant confounders identified in current literature are detailed in Supporting Information S1: Appendix SD [34, 35, 36, 37, 38, 39, 40, 41, 42, 43].

## 3 Results

**Figure 1:**
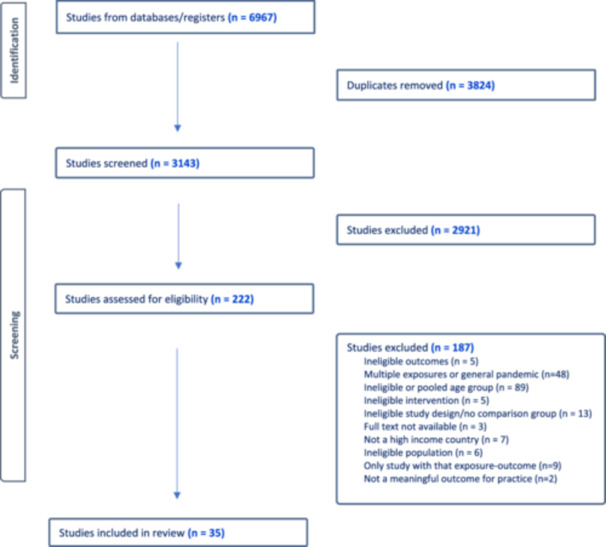
PRISMA results.

Of the 3143 abstracts screened, 222 were relevant for full‐text review and 35 met inclusion criteria for a systematic review (Figure 1).

### 3.1 Study Characteristics

The 35 studies included are detailed in Table 2 [44, 45, 46, 47, 48, 49, 50, 51, 52, 53, 54, 55, 56, 57, 58, 59, 60, 61, 62, 63, 64, 65, 66, 67, 68, 69, 70, 71, 72, 73, 74, 75, 76, 77, 78]. All were non‐randomized cohort studies, representing 14 HIC's, 19 from single sites, 15 across multiple sites, and one including both a single site and statewide sample [58]. All studies, but one [64], had a comparison group unexposed to COVID‐19 policy before the pandemic as opposed to during. All exposure periods were from 2020, capturing a period of high uncertainty, rapid policy changes and no COVID‐19 vaccinations for protective immunity. Regarding COVID‐19 policy exposures, 28 studies focused on lockdowns, five telehealth, one school and business closures, and one bans on gatherings. Sample sizes for the comparison and exposure groups for the populations of interest (mothers, infants, and subgroups where applicable) varied from 8 to 26,924.

**Table 2:** Study characteristics.

Author (year), country	Setting, study design	Study population	Sample size (comparison; exposed)	Data collection period (comparison; exposed)	Policy	Reported outcome categories	OxCGRT range during exposure period daily cases	Covariates (at least 1 outcome was adjusted for)	Lifecourse focus
Alshaikh (2021), Canada [44]	Multicentre, non‐randomized	All live births in the Calgary region. Has 1 positive SARS‐CoV‐2 woman.	24,160; 4357	16 March‐15 June: 2015‐2019; 16 March–15 June: 2020	City lockdown	Preterm, birthweight, stillbirth, hypertensive disorders of pregnancy, gestational diabetes mellitus	24.07 (62.96 by 18 March)‐76.39	Adjusted for seasonality.	Birth
Arias (2022), United States (Pennsylvania) [45]	Single center, non‐randomized	All patients with a postpartum visit of any modality scheduled at the study site between 21 and 56 days postpartum. No mention of the number of participants infected with SARS‐CoV‐2 or if they were excluded.	780; 799	16 March‐30 June: 2019; 16 March‐30 June: 2020	Telehealth (via phone and video)	Breast feeding	N/A	Adjusted for race, prenatal care provider, parity, gestational age at delivery, insurance status, length of hospital stay, hypertensive disorders of pregnancy and seasonality	Postnatal: 21‐56 days
Arnaez (2021), Spain [46]	Multicentre, non‐randomized	All births at the study sites. No mention of the number of participants infected with SARS‐CoV‐2 or if they were excluded.	8710; 1507	15 March‐3 May: 2015‐2019; 15 March‐3 May: 2020	Nationwide lockdown	Preterm, birthweight, stillbirth	67.13‐85.19	Adjusted for hospital, sex, type of delivery, multiple pregnancies, and seasonality.	Birth
Boguslawski (2022), United States (Georgia) [47]	Single center, non‐randomized	All pregnant persons who had at least one prenatal care visit at the study site. 8% of participants were infected with SARS‐CoV‐2.	933; 747	1 March‐31 August: 2019; 1 March‐31 August: 2020	Telehealth (via phone) and home blood pressure monitoring	Antenatal visits, hypertensive disorders of pregnancy, gestational diabetes mellitus, breast feeding	N/A	Adjusted for seasonality.	Antenatal: first, second, and third trimester
Bothara (2021), New Zealand [48]	Single center, non‐randomized	All paediatrics patients (< 16 years) presenting to emergency department at study site. No participants were infected with SARS‐CoV‐2.	209; 136 (for < 1‐year age group)	15 February‐18 March: 2020; 26 March‐28 April: 2020	Nationwide lockdown	Infant ED admissions	87.04‐96.30	Crude	Postnatal: 0‐< 12 months
Cesano (2021), Italy [49]	Single center, non‐randomized	All pregnant women admitted to study site to give birth. 3.9% of participants were infected with SARS‐CoV‐2.	1215; 1260	1 March‐30 April: 2019; 1 March‐30 April: 2020	Nationwide lockdown	Preterm, birthweight, stillbirth, breast feeding	64.35‐93.52	Adjusted for seasonality.	Antenatal:first, second, and third, birth, immediate postnatal
Chaiyachati (2020), United States (Philedelphia) [50]	Single center, non‐randomized	All paediatric patients (< = 21 years) presenting to emergency department at study site. Excluded those left without being seen, transferred to another institution, > 21 years, or left against medical advice. No mention of the number of participants infected with SARS‐CoV‐2.	3838; 531 (for < 1‐year age group)	23 March‐21 April: 2017‐2019; 23 March‐21 April: 2020	Statewide lockdown	Infant ED admissions	72.69	Adjusted for seasonality	Postnatal: 0‐< 12 months
Dopfer (2020), Germany [51]	Single center, non‐randomized	All patients presenting to the emergency department at study site. No mention of the number of participants infected with SARS‐CoV‐2 or if they were excluded.	< 1‐year age group sample size not mentioned	18 March‐14 April: 2019; 16 March‐12 April: 2020	Nationwide lockdown	Infant ED admissions	42.13 (52.31 by 18 March)‐76.85	Adjusted for seasonality	Postnatal: 0‐12 months
Duryea (2021), United States (Texas) [52]	Single center, non‐randomized	All women delivering at study site with birthweight > 500 g. No mention of the number of participants infected with SARS‐CoV‐2 or if they were excluded.	6559; 6048	1 May‐31 October: 2019; 1 May‐31 October: 2020	Telehealth (via phone)	Antenatal visits, hypertensive disorders of pregnancy, gestational diabetes mellitus, stillbirth, preterm.	N/A	Adjusted for body mass index at delivery, race/ethnicity and seasonality.	Antenatal: first, second, and third trimester
Fresson (2022), France [53]	Multicentre, non‐randomized	All delivery hospitalizations to women with a singleton pregnancy at or > 22 weeks in France. No mention of the number of participants infected with SARS‐CoV‐2 or if they were excluded.	3,179,532 (total live births wider period 1 January 2016‐31 July 2020); 96076	5 January 2016‐16 March 2020; 17 March‐10 May: 2020	Nationwide lockdown	Preterm, stillbirth	87.96	Adjusted for maternal age.	Birth
Garabedian (2021), France [54] Quibel (2022), France [55]	Multicentre, non‐randomized	All singleton or multiple pregnancies delivering at > = 24 weeks of gestation with a birthweight > = 500 g. Excluded terminations of pregnancy and home births which led to admission in one of the multiple study sites. 2 and 33 participants hospitalized were infected with SARS‐CoV‐2 comparison and exposed group, respectively.	4093; 3829	22 January‐16 March: 2020; 17 March‐10 May: 2020	Nationwide lockdown	Hypertensive disorders of pregnancy, gestational diabetes mellitus, preterm, birthweight, stillbirth, perinatal mortality	87.96	Adjusted for place of birth, maternal age, multiparity, multiple pregnancies, diabetes, hypertensive disorders, and mode of delivery	Birth
Harvey (2021), United States (Tennessee) [56]	Multicentre, non‐randomized	All births in Tennessee. Excluded gestational age < 17 weeks or > 47 weeks. No mention of the number of participants infected with SARS‐CoV‐2 or if they were excluded.	41,713; 8132	22 March‐30 April: 2015‐2019; 22 March‐30 April: 2020	Statewide lockdown	Hypertensive disorders of pregnancy, gestational diabetes mellitus, preterm, birthweight	72.69	Adjusted for maternal age, education, race/ethnicity, diabetes, hypertension, and seasonality.	Antenatal
Hedley (2022), Denmark [57]	Multicentre, non‐randomized	All singleton births ≥ 22 weeks gestational age. Excluded induced abortions. No mention of the number of participants infected with SARS‐CoV‐2 or if they were excluded.	5230.2 (207.4) mean (SD); 5013	12 March‐14 April: 2015‐2019; 12 March‐14 April: 2020	Nationwide lockdown	Preterm, neonatal mortality, stillbirth	37.96 (62.96 by 13 March)‐72.22	Adjusted for seasonality.	Birth
Jasper (2022), Australia [58]	Single center, non‐randomized Multicentre, non‐randomized	All births at the study site. No mention of the number of participants infected with SARS‐CoV‐2 or if they were excluded. All births in Queensland statewide. No mention of the number of participants infected with SARS‐CoV‐2 or if they were excluded.	1095; 547 20,939; 10,154	1 April‐31 May: 2018‐2019; 1 April‐31 May: 2020	Nationwide lockdown	Preterm, stillbirth	64.43‐73.15	Adjusted for maternal age, BMI, parity and seasonality.	Birth
Keays (2020), Canada [59]	Single center, non‐randomized	All patients presenting to emergency department at study site with motor vehicle collisions, sports‐related injuries, and injuries from recreational activities. No mention of the number of participants infected with SARS‐CoV‐2 or if they were excluded.	3829; 99	16 March‐15 May: 1993‐2019; 16 March‐15 May: 2020	Regional lockdown	Infant ED admissions	43.52‐76.39	Adjusted for seasonality.	Postnatal: 0‐< 12 months
Klumper (2021), Netherlands [60]	Multicentre, non‐randomized	All live births. No mention of the number of participants infected with SARS‐CoV‐2 or if they were excluded.	108,181; 26,924	15 March‐15 May: 2015‐2018; 15 March‐15 May: 2020	Nationwide lockdown	Preterm	53.70‐78.70	Adjusted for seasonality.	Birth
Latorre (2021), Italy [61]	Single center, non‐randomized	Healthy newborns with a gestational age ≥ 37 weeks, “rooming in” (baby in the same room of the mother all day) from birth to discharge and never hospitalized in Neonatal Intensive or Sub‐Intensive Care Unit were included. Excluded were all maternal and/or neonatal conditions that could interfere with breast feeding (maternal HIV or active tuberculosis infection, herpes simplex lesions on both breasts, use of therapeutic radioactive isotopes, or exposure to radioactive materials, galactosemia of the infant) or women not speaking Italian to ensure a full understanding of the questionnaire or not infected with SARS‐CoV‐2.	173; 173	2018; 9 March‐8 May: 2020	Nationwide lockdown	Breast feeding	74.54‐93.52	1:1 matching according to infant's gender, maternal schooling, prenatal class attendance and caesarean section, maternal age	Postpartum: immediate
Leibovitch (2021), Israel [62]	Multicentre, non‐randomized	All live births. Excluded were gestational ages of < 22 or > 44 weeks and multiple pregnancies. No mention of the number of participants infected with SARS‐CoV‐2 or if they were excluded.	77,903; 25,639	11 March‐5 May: 2017‐2019; 11 March‐5 May: 2020	Nationwide lockdown	Preterm	33.33 (62.96 by 15 March)‐94.44	Adjusted for year, seasonality and infant sex.	Birth
Lucero (2020), United States [63]	Multicentre, non‐randomized	All patients presenting to emergency departments at study sites. No mention of the number of participants infected with SARS‐CoV‐2 or if they were excluded.	485,097; 3291	1 January 2017‐pre‐lockdown date for each state 2020; Start of lockdown for each state 2020‐20 April 2020	Statewide lockdown	Infant ED admissions	67.13‐72.69	Crude	Postnatal: 0‐< 12 months
Matei (2021), Romania [64]	Single center, non‐randomized	Pregnant patients aged < 19 years old who gave birth at the study clinic. No participants were infected with SARS‐CoV‐2.	171; 30	16 May‐31 December: 2020; 16 March‐15 May: 2020	Nationwide lockdown	Maternal mental health	58.33‐87.04	Crude	Postnatal: days 2‐3 postpartum
Morgan (2022), United Kingdom [65]	Multicentre, non‐randomized	All births at the study sites. No mention of the number of participants infected with SARS‐CoV‐2 or if they were excluded.	1192.8 (58.8) per week mean (SD); 1090.7 (35.0) per week mean (SD); 1016.7 (60.0) per week mean (SD)	29 December 2014‐22 March 2020; 23 March‐14 June: 2020 (lockdown 1); 9 November 2020‐3 January 2021 (lockdown 2)	Nationwide lockdown	Stillbirth, preterm	67.59‐79.63 (lockdown 1) 63.89‐79.63 (lockdown 2)	Adjusted for trends and variations over time.	Birth
Morris (2021), United States (Southern California) [66]	Single center, non‐randomized	Comparison group was pregnant women expecting their first child, cohabiting, and able to complete study measures in English recruited through social media and word of mouth. Exposed group was expectant parents (any parity) recruited through social media, online pregnancy/parenting groups, and message boards. No mention of the number of participants infected with SARS‐CoV‐2 or if they were excluded.	99; 572	March 2014‐early February 2020; 6 April‐24 May: 2020	Statewide lockdown	Maternal mental health	72.69	Adjusted for race/ethnicity, education, days pregnant, and maternal age.	Antenatal: second and third trimester
Ornaghi (2021), Italy [67]	Single center, non‐randomized	All uninfected and asymptomatically infected women who gave birth at study site.	466; 409	8 March‐18 May: 2019; 8 March‐18 May: 2020	Regional lockdown	Hypertensive disorders of pregnancy, gestational diabetes mellitus, preterm, stillbirth	67.59‐93.52	Adjusted for seasonality.	Antenatal: first, second, and third trimester. Birth
Palmer (2021), Australia [68]	Multicentre, non‐randomized	All births at study site = > 20 weeks’ gestation or with a birthweight = > 400 g if gestation was uncertain. No participants were infected with SARS‐CoV‐2.	20,031; 2292	1 January 2018‐22 March 2020; 20 April‐26 July: 2020	Telehealth (via phone and video) accompanied by automated home blood pressure monitoring and self‐measured symphyseal‐fundal heights	Hypertensive disorders of pregnancy, gestational diabetes mellitus, preterm, birthweight, stillbirth	N/A	Crude	Antenatal: first, second, and third trimester
Pariente (2020), Israel [69]	Single center, non‐randomized	Healthy women with a singleton pregnancy, who delivered a healthy baby > 37 weeks gestation at the study site. No mention of the number of participants infected with SARS‐CoV‐2 or if they were excluded.	123; 223	November 2016‐April 2017; 18 March‐29 April: 2020	Nationwide lockdown	Maternal mental health	77.78‐94.44	Adjusted for maternal age, ethnicity, marital status, and adverse pregnancy outcome.	Postnatal: day 2 postpartum
Silvagni (2021), Italy [70]	Single center, non‐randomized	All paediatric patients presenting to emergency department. No mention of the number of participants infected with SARS‐CoV‐2 or if they were excluded.	622; 262 (for < 1‐year age group)	1 March‐30 April: 2019; 1 March‐30 April: 2020	Nationwide lockdown	Infant ED admissions	64.35‐93.52	Adjusted for seasonality	Postnatal: 0‐< 12 months
Silverman (2020a), United States (New York) [71]	Single center, non‐randomized	All women aged presenting for their first prenatal appointment at the study site. No mention of the number of participants infected with SARS‐CoV‐2 or if they were excluded.	154; 155	2 February‐11 March: 2020; 4 May‐12 June: 2020	Statewide school and non‐essential businesses closure	Maternal mental health	72.69	Crude	Antenatal: first prenatal appointment
Silverman (2020b), United States (New York) [72]	Multicentre, non‐randomized	All women presenting either in person or virtually for their postpartum appointment at one of the study sites. No mention of the number of participants infected with SARS‐CoV‐2 or if they were excluded.	264; 252	2 January‐12 March: 2020; 13 March‐30 June: 2020	Statewide ban on large gatherings	Maternal mental health	30.09 (72.98 by 21 March)	Crude	Postnatal: 6 weeks postpartum
Soffer (2022), United States (Massachusetts) [73]	Single center, non‐randomized	All term singleton non‐anomalous neonates born at study site. 5.1% of participants who had an SGA infant had confirmed SARS‐CoV‐2.	1345; 1296	1 April‐31 July: 2019; 1 April‐31 July: 2020	Telehealth via phone or video (accompanied by changes in the group antenatal testing protocols with decreasing the total number and frequency of ultrasound and provision of home blood pressure monitoring)	Antenatal visits, birthweight	N/A	Adjusted for seasonality	Antenatal: first, second, and third trimester
Soo (2021), Singapore [74]	Single center, non‐randomized	All paediatric outpatients and inpatients at the study site with an orthopaedic injury caused by an acute traumatic event. Excluded for no clear account of a traumatic event resulting in the hospital visit, or cases of isolated limb burns and head injuries. No mention of the number of participants infected with SARS‐CoV‐2 or if they were excluded.	11; 8 (for < 1‐year age group)	7 April‐1 June: 2019; 7 April‐1 June: 2020	Nationwide lockdown	Infant ED admissions	62.04‐82.41	Adjusted for seasonality	Postnatal: 0‐< 12 months
Speyer (2021), United Kingdom [75]	Multicentre, non‐randomized	All births. Excluded if pregnant women were positive for SARS‐CoV‐2.	8323; 7342	24 March‐31 May: 2018; 24 March‐31 May: 2020	Nationwide lockdown	Stillbirth, perinatal mortality, breast feeding	69.44‐79.63	Adjusted for seasonality	Birth
Vacaru (2021), Netherlands [76]	Multicentre, non‐randomized	Comparison group were all pregnant women recruited from an online survey at midwifery centers, ultrasound scan centers, a lactation practice and social media. Excluded incomplete data and women with a complicated pregnancy needing specialized treatment. Exposure group were all pregnant women recruited from an online survey at midwifery centers, word of mouth, social media and other sources. Excluded those who gave a random sequence of answers. No mention of the number of participants infected with SARS‐CoV‐2 or if they were excluded.	1439; 1419	March 2017‐September 2018; 4th April‐10th May: 2020	Nationwide lockdown	Maternal mental health	78.70	Crude	Antenatal: first, second, and third trimester
Zanardo (2021), Italy [77]	Single center, non‐randomized	Women 1 > 8 years, able to read and understand Italian, who had delivered a singleton, healthy neonate at term at the study site. Excluded for prolonged hospital stay, general anaesthesia, undergoing psychological treatment, infant with jaundice, declined to participate or had incomplete data. No mention of the number of participants infected with SARS‐CoV‐2 or if they were excluded.	147; 152	22 February‐18 May: 2019; 22 February‐18 May: 2020	Nationwide lockdown	Maternal mental health breast feeding	37.04 (64.35 by 23 February 2020)‐93.52	Matched (unclear for which variables)	Postnatal: day 2 postpartum
Zanardo (2020), Italy [78]	Single center, non‐randomized	Mothers > 18 years, able to read and understand Italian, delivered a singleton healthy neonate at term at study site. Excluded for general anaesthesia, psychological treatment, hospitalization for SARS‐CoV‐2, infants with jaundice, declined to participate or had incomplete data.	101; 91	8 March‐3 May: 2019; 8 March‐3 May: 2020	Nationwide lockdown	Maternal mental health	74.54‐93.52	Matched (unclear for which variables)	Postnatal: day 2 postpartum

Abbreviations: GDM, gestational diabetes mellitus; HDP, hypertensive disorders of pregnancy.

Table 3 discusses the policy variations for each of the 14 HICs [79, 80, 81, 82, 83, 84, 85, 86, 87, 88, 89, 90, 91, 92]. In addition to this is Supporting Information S1: Appendix SE which shows the OxCGRT Stringency Index scores across the countries and how they varied over each studies exposure period (highlighted in the gray rectangle). Both Table 3 and Supporting Information S1: Appendix SE confirm that all included studies had a high OxCGRT Stringency Index for their exposure periods, validating our strict definition of exposure periods within natural experiments to closely emulate an RCT for casual inference. Of the 30 studies that focused on lockdowns, bans on gathering, or closures of school and businesses, 80% (24/30) had a minimum OxCGRT Stringency Index score above 50 out of 100 for the duration of the exposure period and the remaining 20% (6/30) had a minimum score above 50 within 1–7 days of the exposure period, e.g., in Hedley (2020)'s study, the exposure period was between March 12 and April 14, 2020 and the minimum OxCGRT score was 37.96 but up to 62.96 within one day.

**Table 3:** Policy variation across countries and their corresponding studies.

Country	Lockdown	Author (year) Policy exposure period OxCGRT minimum‐ maximum during policy exposure period	Telehealth	Author (year) Policy exposure period
Australia	National and regional lockdowns with early, strict border closures [79]. A nationwide lockdown between March to May 2020. State‐based restrictions continued throughout 2020. Closed to international travel from March 2020 which continued throughout 2020 with quarantine mandates.	Jasper (2022) 1 April‐31 May: 2020 64.43‐73.15	Telehealth requirements varied across health services [80]. i. For this study, telehealth was via phone and video accompanied by automated home blood pressure monitoring and self‐measured symphyseal‐fundal heights.	i. Palmer (2021) 20 April‐26 July: 2020
Canada	Regional lockdowns with early, strict border closures [81]. No nationwide lockdown was implemented. Province‐based lockdowns began in March 2020 and varied throughout 2020. Closed to international travel from March 2020 which continued throughout most of 2020 with quarantine mandates.	Alshaikh (2021) 16 March‐15 June: 2020 24.07 (up to 62.96 within 2 days)‐76.39 Keays (2020) 16 March‐15 May: 2020 43.52‐76.39		
Denmark	National lockdowns with early border closures but early reopening to European Union (EU) countries [82, 83, 84]. A nationwide lockdown began in March 2020 with phased reopening in April 2020. Further partial lockdown in December which continued into 2021. Closed to international travel from March to June 2020.	Hedley (2022) 12 March‐14 April: 2020 37.96 (up to 62.96 within 1 day)‐72.22		
France	National lockdowns with early border closures but early reopening to EU countries [84, 85]. A nationwide lockdown between March and May 2020 and October and December 2020. Closed to international travel from March 2020 except for essential travel with reopening in June.	Fresson (2022) 17 March‐10 May: 2020 87.96 Garabedian (2021) 17 March‐10 May: 2020 87.96 Quibel (2022) 17 March‐10 May: 2020 87.96		
Germany	National and regional lockdowns with early border closures but early reopening to EU countries [84, 86]. A nationwide lockdown began in March to May 2020 and in November and continued throughout 2020. Local lockdowns throughout 2020. Borders closed in March 2020 and reopened in June 2020.	Dopfer (2020) 16 March‐12 April: 2020 42.13‐76.85		
Israel	National lockdowns with early, strict border closures [87]. A nationwide lockdown between March to May, September to October and December 2020. International travel is banned from March and remains that way throughout 2020.	Leibovitch (2021) 11 March‐5 May: 2020 33.33 (up to 62.96 within 4 days)‐94.44 Pariente (2020) 18 March‐29 April: 2020 77.78‐94.44		
Italy	National lockdowns with early border closures, but early reopening to EU countries [84, 88]. A nationwide social distancing restriction rather than strict lockdown from March to May 2020 and November through to 2021. Closed borders in March to June 2020.	Cesano (2021) 1 March‐30 April: 2020 64.35‐93.52 Ornaghi (2021) 8 March‐18 May: 2020 67.59‐93.52 Latorre (2021) 9 March‐8 May: 2020 74.54‐93.52 Silvagni (2021) 1 March‐30 April: 2020 64.35‐93.52 Zanardo (2021) 22 February‐18 May: 2020 37.04 (up to 64.35 within 1 day)‐93.52 Zanardo (2020) 8 March‐3 May: 2020 74.54‐93.52		
Netherlands	National lockdown with early border closures but early reopening to EU countries [89]. A nationwide ‘intelligent lockdown’ lockdown which had allowed people to still leave their homes but with social distancing was implemented from March to May 2020 and a further partial lockdown in October 2020. Closure of borders from March to June 2020.	Klumper (2021) 15 March‐15 May: 2020 53.70‐78.70 Vacaru (2021) 4 April‐10 May: 2020 78.70		
New Zealand	National and regional lockdowns with early, strict border closures [90]. A nationwide lockdown was implemented from March to May 2020 followed by city‐based lockdowns in August to September 2020. International border closures throughout 2020 requiring quarantine.	Bothara (2021) 26 March‐28 April: 2020 87.04‐96.30		
Romania	National lockdown with early border closures, but early reopening to EU countries [93]. A nationwide lockdown was implemented from March to May 2020 followed by partial lockdowns in November which continued into 2021. Closures of borders in March to June 2020.	Matei (2021) 7 April‐1 June: 2020 62.04‐82.41		
Singapore	Targeted and adaptive national lockdown with early, strict border closures [94]. A national ‘circuit breaker’ lockdown from April to June 2020 focusing on strict public health controls rather than prolonged lockdowns. International border closures with phased reopening August 2020.	Soo (2021) 16 March‐15 May: 2020 58.33‐87.04		
Spain	National and regional lockdown with early border closures but early reopening to EU countries [84, 91]. A nationwide lockdown was implemented from March to May 2020 and return to regional lockdowns in October 2020. Closure of borders in March until July 2020.	Arnaez (2021) 15 March‐3 May: 2020 67.13‐85.19		
United Kingdom	National lockdown with travel restrictions but no full border closures [92]. A nationwide lockdown was implemented from March to June 2020. Phased reopening over summer followed by a return to lockdown in November to December 2020. The UK never fully closed its borders.	Morgan (2022) 23 March‐14 June: 2020 (lockdown 1) 9 November 2020‐3 January 2021 (lockdown 2) 67.59‐79.63 (lockdown 1) 63.89‐79.63 (lockdown 2) Speyer (2021) 24 March‐31 May: 2020 69.44‐79.63		
United States of America	Regional lockdowns with travel restrictions but no full border closures [95]. No national lockdown: state and local governments implemented lockdowns from March 2020 with most lifting restrictions by June 2020. The USA never fully closed its borders.	Chaiyachati (2020) 23 March‐21 April: 2020 72.69 Harvey (2021) 22 March‐30 April: 2020 72.69 Lucero (2020) Start of lockdown for each state until 20 April 2020 67.13‐72.69 Morris (2021) 6 April‐24 May: 2020 72.69 Silverman (2020a) 4 May‐12 June: 2020 72.69 Silverman (2020b) 13 March‐30 June: 2020 30.09 (up to 72.98 within 7 days)	Telehealth requirements varied across health services [96]. i. For this study, telehealth was via phone and video ii. For this study, telehealth was via phone accompanied by home blood pressure monitoring iii. For this study, telehealth was via phone iv. For this study, telehealth was via phone or video accompanied by home blood pressure monitoring and an overall decrease in testing protocols with less ultrasounds.	i. Arias (2022) 16 March‐30 June: 2020 ii. Boguslawski (2022) 1 March‐31 August: 2020 iii. Duryea (2021) 1 May‐31 October: 2020 iv. Soffer (2022) 1 April‐31 July: 2020

Across the five studies focusing on telehealth as the exposure policy, two studies were telehealth as phone appointments only whilst three were telehealth as both phone and video appointments. Three of these telehealth studies in addition provided at home blood pressure monitoring.

On the NOS, 5 studies scored “'Low risk”, 18 “Moderate risk”, and 12 “High risk” in quality (Table 4). The main weaknesses across studies were no adjustments for confounders and no reporting on missing data. Only 15 of the 35 studies adjusted or matched for confounders other than seasonality, and 19 studies considered seasonality by comparing groups within the same months but different years.

**Table 4:** Study results for Newcastle‐Ottawa Scale.

Author (year), Country	Representativeness of the exposed group	Selection of the non‐exposed group	Ascertainment of the exposure	Demonstration that outcome of interest was not present at start of study	Comparability of groups on the basis of the design or analysis	Comparability of groups on the basis of the design or analysis2	Assessment of outcome	Was follow up long enough	Adequacy of follow up of cohorts	Risk of bias
Fresson (2022), France	*	*	*	*	*		*	*	*	Low
Garabedian (2021), France	*	*	*	*	*	*	*	*	*	Low
Harvey (2021), United States (Tennessee)	*	*	*	*	*	*	*	*		Low
Jasper (2022), Australia	*	*	*	*	*	*	*	*		Low
Quibel (2022), France	*	*	*	*	*	*	*	*	*	Low
Alshaikh (2021), Canada	*	*	*	*		*	*	*		Moderate
Arias (2022), United States (Pennsylvania)	*	*	*	*		*	*	*		Moderate
Arnaez (2021), Spain	*	*	*	*		*	*	*		Moderate
Boguslawski (2022), United States (Atlanta, Georgia)	*	*	*	*		*	*	*		Moderate
Cesano (2021), Italy	*	*	*	*		*	*	*		Moderate
Chaiyachati (2020), United States (Philedelphia)	*	*	*	*		*	*	*		Moderate
Dopfer (2020), Germany	*	*	*	*		*	*	*		Moderate
Duryea (2021), United States (Texas)	*	*	*	*		*	*	*		Moderate
Hedley (2022), Denmark	*	*	*	*		*	*	*		Moderate
Keays (2020), Canada	*	*	*	*		*	*	*	*	Moderate
Klumper (2021), Netherlands	*	*	*	*		*	*	*	*	Moderate
Leibovitch (2021), Israel	*	*	*	*		*	*	*		Moderate
Morgan (2022), United Kingdom‐	*	*	*	*		*	*	*	*	Moderate
Ornaghi (2021), Italy	*	*	*	*		*	*	*		Moderate
Silvagni (2021), Italy	*	*	*	*		*	*	*		Moderate
Soffer (2022), United States (Massachusetts)	*	*	*	*		*	*	*		Moderate
Soo (2021), Singapore	*	*	*	*		*	*	*		Moderate
Speyer (2021)	*	*	*	*		*	*	*		Moderate
Bothara (2021), New Zealand	*	*	*	*			*	*		High
Latorre (2021), Italy	*	*	*	*	*	*		*		High
Lucero (2020), United States	*	*	*	*			*	*		High
Matei (2021), Romania	*	*	*					*	*	High
Morris (2021), United States (Southern California)	*		*		*	*		*		High
Palmer (2021), Australia	*	*	*	*			*	*	*	High
Pariente (2020), Israel	*	*	*		*	*		*		High
Silverman (2020a), United States (New York)	*	*	*					*	*	High
Silverman (2020b), United States (New York)	*	*	*					*	*	High
Vacaru (2021), Netherlands	*		*					*		High
Zanardo (2021), Italy	*	*	*					*	*	High
Zanardo (2020), Italy	*	*	*					*	*	High

*Note:* A star (*) assigned in accordance with the Newcastle‐Ottawa Scale scoring criteria, as detailed in Appendix SC of the Supplementary Files.

### 3.2 Key Findings

Lockdown was associated with decreases in preterm and low birthweight births, fewer infant emergency admissions, and a lower number of breast feeding women, and associated with increases in hypertensive disorders of pregnancy and gestational diabetes mellitus. Telehealth was associated with younger gestational age at initial antenatal visits and a lower number of breast feeding women. There were mixed results for maternal mental health and stillbirths.

**Figure 2:**
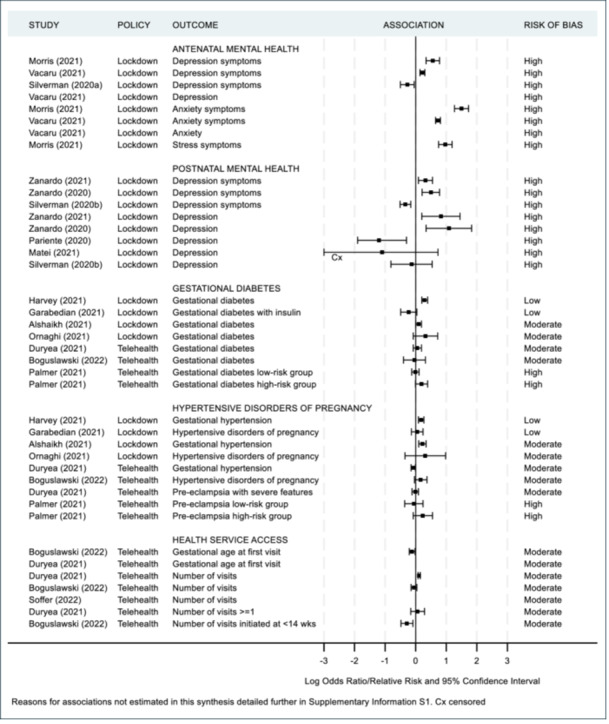
Maternal health outcomes.

**Figure 3:**
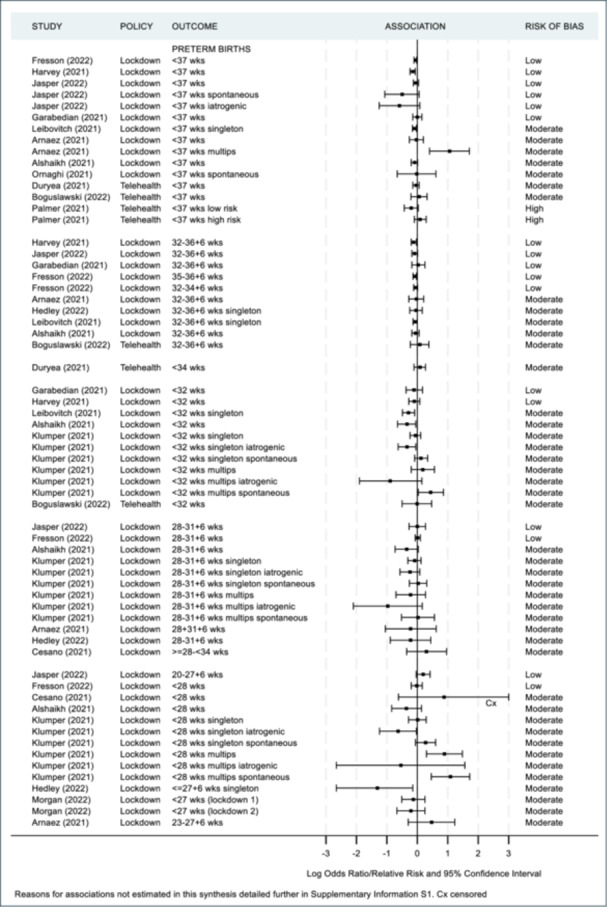
Infant health outcomes ‐ preterm births.

**Figure 4:**
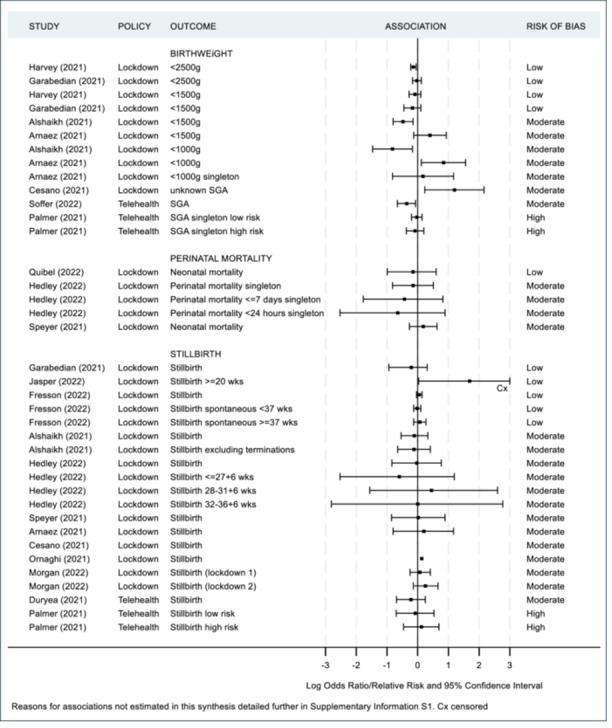
Infant health outcomes ‐ birthweight, perinatal mortality and stillbirth.

**Figure 5:**
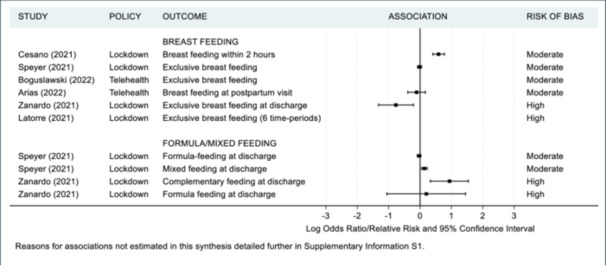
Infant health outcomes ‐ nutrition.

**Figure 6:**
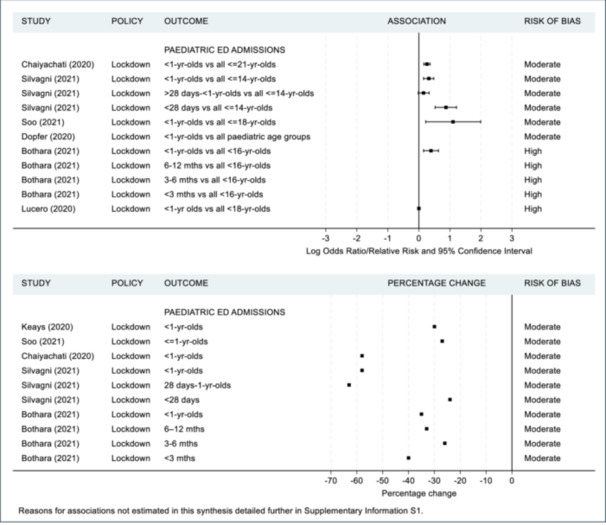
Infant emergency department admissions compared to all paediatric age‐groups during the pandemic (top) and infants prior to pandemic (bottom).

As previously mentioned, a meta‐analysis was not appropriate in accordance with the Cochrane guidelines [19] since the majority of studies were of moderate to high risk of bias. Additionally there was substantial contextual diversity (differences in participant demographics, i.e., under 37 weeks iatrogenic vs. under 37 weeks singleton); methodological diversity (differences in measurement tools used, i.e., different questionnaires used to assess maternal mental health, differences in cut off points for clinically meaningful scores such as the variety in age‐group range for paediatric ED admissions); and statistical diversity (differences in between group estimates with odds ratios, risk ratios and rate ratios provided), once again preventing meta‐analyses. Instead, maternal and infant health outcomes are summarized in Figures 2, 3, 4, 5 and 6, including visual display of the patterns of the evidence. Supporting Information S1: Appendix SF includes further details of the extracted numerical data. There was no clear pattern of the prevalence of SARS‐CoV‐2 in study regions having an association with maternal and infant outcomes, although, included studies reported low infection rates or excluded women and infants who had SARS‐CoV‐2 infections (Table 2).

### 3.3 Maternal Mental Health

Containment and closure policies (lockdown, school, and non‐essential business closures or bans on large gatherings) and maternal mental health were examined in eight studies, all at high risk of bias and with mixed results. Four screening tools for anxiety and depression were used across these studies [64, 66, 69, 71, 72, 76, 77, 78]. In the four studies with underserved communities (ethnic minority groups ‐ Hispanic, Black, Bedouin; single, unmarried adolescents; or socioeconomically disadvantaged) [64, 69, 71, 72], decreases in symptoms of mental illness were reported with effect sizes ranging from −0.27 to −1.20. In contrast, the other four general population studies reported increases in symptoms of mental illness during lockdown with effect sizes ranging from 0.22 to 1.50. Participants from these studies were mostly married or had high levels of education [66, 76, 77, 78].

### 3.4 Maternal Physical Health

Four studies examined the associations of containment and closures with hypertensive disorders of pregnancy and gestational diabetes mellitus [44, 54, 56, 67]. Two were moderate risk of bias [44, 67] and two low‐risk [54, 56]. All studies reported an increase in hypertensive disorders of pregnancy during lockdown (RR/OR between 1.05 and 1.37), and three reported an increase of gestational diabetes mellitus during lockdown (OR between 1.10 and 1.37). The three studies that examined associations of telehealth with hypertensive disorders of pregnancy and gestational diabetes mellitus showed no consistent direction of change [47, 52, 68].

### 3.5 Maternal Health Service Access

Three studies examined the association of telehealth and antenatal health service use [47, 52, 73]. All were moderate risk of bias and despite these studies focusing on telehealth as the policy exposure, almost a quarter to over half of the exposed groups only attended in‐person appointments. Findings were mixed, with a pattern suggesting increased antenatal health service use, particularly at earlier gestations, when compared to the period before introduction of telehealth as a general policy.

### 3.6 Preterm and Low Birthweight

Twelve studies examined the association of lockdown and preterm births with four being low risk of bias [54, 56, 58], and eight moderate‐risk [44, 46, 49, 53, 57, 60, 62, 65, 67]. Studies were divided into subgroups based on gestational age and compared to the reference group of all births unless specified otherwise. Seven of eight studies assessing all or singleton preterm (< 37 weeks) reported a decrease (OR/RR between 0.56 and 0.98) [44, 46, 53, 56, 58, 62, 67] and seven of eight studies assessing all or just singleton late preterms (32–36 + 6 weeks) reported a decrease (OR between 0.89 and 0.97) [44, 46, 53, 56, 57, 58, 62]. There was no consistent change in very preterm (28–31 + 6 weeks) or extreme preterm (<28 weeks) births. Of the twelve preterm studies, five also examined the association of lockdown and low birthweight or small for gestational age (SGA) with two being of low risk of bias [54, 56] and three moderate‐risk [44, 46, 49]. Three of the five studies reported small decreases in low birthweight suggesting higher weights of infants born during lockdown (OR/RR between 0.44 and 0.97) [44, 54, 56]. The four studies that examined the association of telehealth showed an inconsistent pattern of change for preterm births [47, 52, 68, 73].

### 3.7 Stillbirth

Ten studies examined the exposure of lockdown and stillbirth with three being low risk of bias [54, 58] and seven moderate‐risk [44, 46, 49, 53, 57, 65, 67, 75]. All births were used as the reference group unless specified otherwise. There was an inconsistent pattern of change with wide 95% CI's likely due to small sample numbers given the infrequency of this outcome.

### 3.8 Paediatric Emergency Department (ED) Admissions

Seven studies examined the associations of lockdown with ED admissions with five being moderate risk of bias [50, 51, 59, 70, 74] and two high‐risk [48, 63]. Of the five studies that reported on numbers of < 1 year old admissions to ED during lockdown, all of them reported a decrease between 24% and 63%. Figure 6 outlines that of the six studies which examined the proportion of < 1‐year‐olds ED admissions compared to other paediatric age groups [48, 50, 51, 63, 70, 74], five reported an increase (OR between 1.17 and 3.02) [48, 50, 51, 70, 74].

### 3.9 Breast Feeding

Four studies examined the association of lockdown and breast feeding, with two being moderate risk of bias [49, 75] and two high‐risk [61, 77]. Three of the four studies reported either a decrease in the number of breast feeding women during lockdown (OR between 0.46 and 0.99, or 28% reduction) [61, 75, 77] or an increase in the number of women feeding with formula (OR between 1.15 and 2.56) [75, 77]. Two studies examined the association of telehealth appointments and breast feeding [45, 47]. Both these studies were moderate risk of bias and reported a decrease in the number of breast feeding women.

## 4 Comment

This systematic review summarizes the association of specific COVID‐19 policies on maternal and infant outcomes in HIC's. It focuses on policies before the distribution of COVID‐19 vaccinations, a period of rapid change and uncertainty. No RCT's met the inclusion criteria, highlighting the importance, as recognized by Cochrane, of natural experiments to evaluate population‐level policy interventions that are unlikely to be randomized [19].

### 4.1 Principal Findings

Synthesis of 35 studies suggested containment and closure policies were associated with positive perinatal outcomes: decreases in preterm and low birthweight births (notably late preterm); fewer infant ED admissions; and less mental illness in underserved communities. Containment and closure policies were also associated with negative perinatal outcomes: increases in mental illness in higher socioeconomic groups; higher rates of maternal hypertensive disorders of pregnancy and gestational diabetes mellitus; increases in the proportion of infant ED admissions when compared to other paediatric age groups; and a lower number of breast feeding women. Telehealth had a positive outcome of increased engagement at antenatal visits in the first trimester, but a negative outcome of a lower number of breast feeding women. There was no clear direction of change for stillbirths during lockdown.

### 4.2 Strengths of the Review

Several systematic reviews have examined maternal and infant health during the COVID‐19 pandemic, often focusing on biological exposures such as SARS‐CoV‐2 infection or vaccination or assessing outcomes across the general pandemic period without isolating specific policies. In contrast, our review focuses on COVID‐19 policy responses as exposures, such as lockdowns, service closures, and telehealth, that represent structural changes to the social determinants of health. By applying strict inclusion criteria to ensure clearly defined, time‐bound policy exposures and by focussing on outcomes spanning the whole perinatal period for mothers and infants, our review offers a unique contribution.

We synthesized COVID‐19 evidence to avoid interpretation or action based on a single study in isolation. This is important given concerns of scientific integrity of publications by‐passing rigorous pre‐pandemic peer‐review processes [97, 98]. Our comprehensive review followed PRISMA and Cochrane guidelines with two independent researchers. A well‐defined protocol was registered a priori for replicability and transparency. Studies were thoroughly quality‐assessed using the Newcastle‐Ottawa Scale. Strict adherence to COVID‐19 policy exposure timeframes ensured a clear distinction between the the exposed and comparison groups, thus emulating the methodology of an RCT as closely as possible. The fidelity of this method was validated by high OxCGRT Stringency Index across included studies for their exposure periods.

### 4.3 Limitations of the Review

This review has several limitations. Multiple forms of heterogeneity amongst the included studies made meta‐analysis inappropriate. Heterogeneity included contextual (differences in participant demographics, lifecourse timing of exposures); methodological (differences in study design, measurement tools, cut off points for clinically meaningful scores); and statistical (differences in between group estimates). Many findings have wide 95% CI's that have crossed the null value, limiting interpretation of size and direction of change. Studies were retrospective, non‐randomized with only a small number adjusting for confounders other than seasonality or addressing missing data, resulting in moderate to high risk of bias. The review captures only immediate policy associations, missing potential delayed or long‐term impacts. Notably, there was an absence of comparable studies on child developmental outcomes which may be reflected in future upcoming research as the “COVID‐19 generation” reaches school‐age. Whilst a broad range of COVID‐19 policies were searched, included studies focussed only on containment and closure policies (e.g., lockdown) and public health measures (e.g., telehealth), none focused on economic policies (e.g., welfare subsidies, medical insurance extensions and offsets in rent and mortgage repayments). Lastly, whilst exposures were limited to only one COVID‐19 policy per study, it is likely there were multiple unmeasured COVID‐19 policies acting concurrently on participants. Studies did not adjust analyses to account for concurrent COVID‐19 policies, consequences of other COVID‐19 policies, or unmeasured confounders. For example, studies focussing on lockdown adjusted for neither concurrent COVID‐19 financial supports nor the reduction in air pollution, both of which could have an effect on maternal and infant health outcomes.

### 4.4 Hypotheses for Exposure‐Outcome Associations

The findings provide associations of policy changes and associated transformations in maternal‐infant health outcomes. Whilst these associations are not causative, they provide important pathways to pursue future high‐quality causal study designs evaluating pandemic policy. Identifying how structural policy changes may meaningfully shift maternal‐infant outcomes, has important implications for global health equity. Such insights can inform the design of future public health responses and contribute to progressing the achievement of the 2030 Sustainable Development Goal (SDG) 3: “*to ensure healthy lives and promote wellbeing for all at all ages*” (Box 1) [6]. The SDG's offer a valuable framework for contextualizing our results, aligning with recent calls for high‐income countries to prioritize science that supports both domestic and global progress towards these objectives [99].

Box 1 **2030 Sustainable Development Goal targets relevant to maternal and infant health.**

**Table:** 

**2030 Sustainable Development Goal targets** [6]
3.2. To end preventable deaths of newborns and children under 5 years of age 3.3. To end epidemics of communicable diseases 3.4. To reduce global maternal non‐communicable diseases through prevention and treatment and promoting mental health 3.7. To ensure universal access to reproductive healthcare services

Preterm birth and associated complications are the leading cause of death in under 5‐year‐olds [100]. One in 10 infants are preterm ‐ a rate that has remained stagnant for the past decade [101, 102]. Our findings of decreased preterm birth rates during lockdown periods with no clear change in stillbirths are consistent with systematic reviews from HIC's [7, 10], potentially explained by changes in environmental exposures during lockdown. With physical distancing, school and daycare closures and hygiene practices such as mask‐wearing during lockdowns, many viral respiratory infection rates reduced in both the northern and southern hemispheres [103]. Reductions in economic activity and transport saw a global reduction in air pollution during lockdown [104]. Both communicable diseases and air pollution are well‐established risk factors for preterm births [40, 105]. Reduced late preterm births may also reflect decreased access to health services for iatrogenic preterm births [106], though findings lacked the depth for detailed analysis. Additionally, we may underestimate the reduction in preterm births due to SARS‐CoV‐2 infection, which is a risk factor for preterm births [107]. However, this is unlikely as the relevant studies reported infection rates of only < 1%‐4% among participants. These findings offer compelling insights for future studies aimed at advancing Target 3.2 “*to end preventable deaths of newborns and children under 5 years of age*.”

The decrease in infant ED admissions during lockdown is consistent with pre‐COVID‐19 studies on other infectious disease outbreaks [108, 109, 110]. Findings did not allow for comparisons in the reasons for infant admission, however, similar to the reduction in preterm births, fewer infectious diseases may have contributed less infant ED admissions during lockdown [111]. Reductions in childhood communicable disease is consistent with other systematic reviews exploring the general pandemic period [12, 15] Fewer infant ED admissions during lockdown may reinforce evidence towards the promotion of basic public health practices in reducing communicable diseases aligned with progressing Target 3.3 “*to end epidemics of communicable diseases*”. When compared to other paediatric age groups, however, the higher proportion of infants admitted to ED may reflect the limited access to community primary health services during lockdown. These services typically address common infant concerns, such as feeding difficulties, which still require timely support [70, 112, 113]. Findings emphasize the importance of the continuation of primary health services in future pandemics to reduce preventable ED admissions amongst infants.

Mental illness is a leading cause of maternal morbidity and mortality [114]. Evidence from other systematic reviews identified lockdown having overall negative associations with perinatal mental illness with highly educated cohorts experiencing more anxiety, although most of the included studies were cross‐sectional surveys without unexposed comparison groups [16, 17]. Our review builds on previous literature that suggests that mental health may vary depending on sociodemographic characteristics including access to socioeconomic assets [115, 116]. Our review may therefore actually reflect concurrent government economic stimulus packages during lockdowns, such as the provision of COVID‐19 unemployment payments in the countries of studies with positive mental health outcomes [117]. This provides possible avenues for shifting maternal mental health outcomes using transformative policy interventions to advance Target 3.4 “*to reduce global maternal non‐communicable diseases through prevention and treatment and promoting mental health*” [118, 119]. An alternative explanation could be the underreporting of mental illness in underserved populations driven by complex social and service contexts including the impacts of stigma and systemic racism [116].

In addition to maternal mental illness, hypertensive disorders of pregnancy and gestational diabetes mellitus also contribute to a large global burden of non‐communicable disease in pregnant women. Pre‐pandemic, the global incidence of gestational diabetes mellitus was on the rise, but the incidence of hypertensive disorders of pregnancy was falling [120, 121]. Findings in this review suggest lockdown being associated with increased rates of both. These findings are similar to a multicentre study in a location with prolonged lockdowns [122], but contradict a 2021 meta‐analysis reporting no change during the broad pandemic period, indicating that perhaps factors within lockdown contributed to harm [7]. With obesity being a risk factor for hypertensive disorders of pregnancy and gestational diabetes mellitus, increases in body weight during lockdowns noted in Bakaloudi et al (2022) [123] systematic review may explain this especially given the reductions in work commutes, closures of exercise facilities and potential behavioral changes. This is in conjunction with reductions to in‐person antenatal check‐ups. SARS‐CoV‐2 infection is also associated with hypertensive disorders of pregnancy, although an unlikely explanation in this review as the relevant studies reported a below 1% infectivity rate [124].

Telehealth showed early evidence of improving first trimester antenatal engagement. Maternal‐infant health services are vital in reducing perinatal morbidity and mortality as highlighted in the SDG Target 3.7 “*to ensure universal access to reproductive healthcare services*”. This may provide early information on accessibility of telehealth in HIC's. However, both telehealth and lockdowns were associated with decreases in the number of breast feeding women. Support from healthcare services is essential to help mothers initiate and maintain exclusive breast feeding according to the World Health Organization's “Ten Steps to Successful Breast feeding” [125, 126] and disruptions to in‐person breast feeding support may have contributed to the lowered rates of breast feeding and higher uptake in formula feeding [127].

Synthesis of multiple natural experiments provides indicative evidence of policies being transformative in shifting previously stagnant maternal‐infant health parameters. No randomized studies met the inclusion criteria, highlighting the importance of natural experiments, whilst acknowledging limitations in the extent to which confounding factors can be accounted for. Whilst we offer possible evidence‐based interpretations of why COVID‐19 policies may have been associated with shifts in maternal‐infant outcomes, what is needed is rigorous trials. Emerging modeling simulations allow this to be done virtually, such as that being implemented in the longitudinal whole‐of‐state birth cohort study Generation Victoria in Australia (GenV) [128]. This offers opportunities to test hypotheses on policy impact using robust large scale quantitative evaluation [129].

Future pandemic preparedness necessitates improved and more intentional policy comparison on a global‐scale. The standardization of rigorous study designs and the improvement in national routine data collection across countries would improve future policy comparisons amidst global crisis by providing less heterogeneity and lower risk of bias, both barriers to conducting a meta‐analysis within this review. We echo other global calls for this improvement in perinatal data availability [10]. The COVID‐19 pandemic showed our interconnectedness as global citizens and this interconnectedness should be reflected in our research collaboration to maximize policy evaluation and advancement of maternal‐infant health. This would foster the development of the OECD's strategic vision for achieving the 2030 SDG's in an integrated and coherent manner [130].

## 5 Conclusion

This review highlights that COVID‐19 policies, notably lockdowns and telehealth, were associated with both beneficial and adverse maternal‐infant health outcomes. By synthesizing natural experiments, our findings emphasize the potential of transformative policy in shifting maternal‐infant targets relevant to the 2030 Sustainable Development Goals. We (i) call for a broader range of underrepresented COVID‐19 policies, such as economic interventions, to be evaluated alongside lockdowns and telehealth within high‐quality causal study designs, (ii) highlight the need for evaluation of the broader, unintentional effects of pandemic policy on long‐term child developmental outcomes, and (iii) emphasize the urgent need for international collaboration, improved nationwide data sharing, and more rigorous causative study designs to enable meaningful policy comparisons in future pandemics.

## Author Contributions

**Ashleigh Shipton:** conceptualization, data curation, formal analysis, funding acquisition, investigation, methodology, project administration, validation, visualization, writing – original draft, writing – review and editing. **Katie Mcbain:** data curation, validation, writing – review and editing. **Melissa Wake:** conceptualization, funding acquisition, methodology, supervision, writing – review and editing. **Sharon Goldfeld:** conceptualization, funding acquisition, methodology, supervision, writing – review and editing. **Fiona Mensah:** conceptualization, data curation, formal analysis, funding acquisition, investigation, methodology, supervision, validation, visualization, writing – review and editing.

## Disclosure

All authors have read and approved the final version of the manuscript. Dr Fiona Mensah and Dr Ashleigh Shipton had full access to all of the data in this study and take complete responsibility for the integrity of the data and the accuracy of the data analysis.

## Ethics Statement

Ethics approval was received from the Royal Children's Hospital Human Research Ethics Committee (87751) for this review as part of a broader PhD project titled “Looking back to move forward: The effects of the COVID‐19 pandemic and policy responses on women and children's health and development in Victoria, Australia” funded by University of Melbourne, Royal Australasian College of Physicians, and Murdoch Children's Research Institute.

## Consent

The authors have nothing to report.

## Conflicts of Interest

The authors declare no conflicts of interest.

## Transparency Statement

The lead author Fiona Mensah affirms that this manuscript is an honest, accurate, and transparent account of the study being reported; that no important aspects of the study have been omitted; and that any discrepancies from the study as planned (and, if relevant, registered) have been explained.

## Supporting information


**Appendix SA:** Search terms. **Appendix SB:** Data extraction template. **Appendix SC:** Decision rules for application of the Newcastle‐Ottawa Scale. **Appendix SD:** Confounders applicable to the Newcastle‐Ottawa Scale. **Appendix SE:** OxCGRT Stringency Index scores across 14 countries. **Appendix SF:** Maternal and infant health outcomes.

## Data Availability

The study protocol, search strategy, list of included and excluded studies, data extracted, and quality assessment are available in the article and upon request from the corresponding author.
