# Supplementary material for: Policy Responses to the COVID‐19 Pandemic in High‐Income Countries and the Associated Maternal‐Infant Health Outcomes: A Systematic Literature and Policy Review
Source: Health Sci Rep. 2026 May 15;9(5):e71523. doi: 10.1002/hsr2.71523 (PMC13176782; doi:10.1002/hsr2.71523)
Supplement: Supplementary file 1 — Appendix SA: Search terms. Appendix SB: Data extraction template. Appendix SC: Decision rules for application of the Newcastle‐Ottawa Scale. Appendix SD: Confounders applicable to the Newcastle‐Ottawa Scale. Appendix SE: OxCGRT Stringency Index scores across 14 countries. Appendix SF: Maternal and infant health outcomes. [file HSR2-9-e71523-s001.pdf]

Appendix SA - Search terms

Database	COVID-19	Policies	Maternal and infant	Study design
**Ovid MEDLINE**	*COVID-19/ or *SARS-CoV-2/ OR (2019-novel or 2019nCoV or 2019-nCoV or COVID-19 or COVID19 or COVID-2019 or COVID2019 or CONVID-19 or CONVID19 or CORVID-19 or CORVID19 or CoV2 or CoV-2 or HCoV* or Ncov* or Ncorona* or Ncorono* or NcovChina* or NcovChinese* or NcovHubei* or NcovWuhan* or SARS2 or SARS-2 or SARScoronavirus2 or SARScoronavirus-2 or SARScoronovirus2 or SARScoronovirus-2 or SARSCov19 or SARSCov-19 or SARS-CoV-2 or SARSCoV-2 or SARSCoV2 or WN-CoV or WNCoV or wuhan-virus).tw,kf,ot. OR ((pneumonia* or outbreak* or respiratory-illness* or respiratory-disease* or respiratory-symptom* or seafood-market* or food-market* or wildlife) and (Wuhan or China or Chinese or Hubei or Huanan)).tw,kf,ot. OR ((new or novel or nouveau or "19" or "2019" or Wuhan or Hubei or Huanan or China or Chinese) adj3 (coronavirus* or corona virus* or betacoronavirus* or CoV or HCoV)).tw,kf,ot. OR (longCOVID* or postCOVID* or postcoronavirus* or postSARS*).ti,ab,kf,ot. OR (*coronavirus/ or *betacoronavirus/ or *coronavirus infections/) and (*disease outbreaks/ or *epidemics/ or *pandemics/) OR ((coronavirus* or corona-virus* or betacoronavirus*) adj3 (pandemic* or epidemic* or outbreak* or crisis)).tw,kf,ot	fiscal policy/ or public policy/ or health policy/ or health care reform/ OR (Lockdown* or lock-down* or closure* or containment* or restriction* or density-limit* or cancel* or postpone* or quarantine* or isolation or stay-at-home* or close-contact* or household-contact* or redeploy* or JobKeeper* or JobSeeker* or unemploy* or subsid* or income* or tax-relief* or mortgage-relief* or rent-relief* or financial or economic* or cash-transfer* or cash-payment or allowance* or paid-sick-leave or travel* or radius or childcare* or child-care* or government-support* or welfare* or social-security* or mass-gathering* or social-distanc* or physical-distanc* or mask* or visitor-restriction* or public-health-measure* or government-regulation*).tw,kf. OR (telemedicine or tele-medicine or remote-monitor* or long-distance-monitor* or remote-sensing or telesensing or tele-sensing or telemonitoring or tele-monitoring or telehealth or tele-health or teleconsultation* or tele-consultation* or virtual-care or virtual-health or mobile-health or m-health or app or apps or e-health or ehealth or telepractice or tele-practice or technology-based or emerging-technolog* or digital-health).tw,kf. OR (policy or policies or government-order? or mandate*).tw,kf. OR physical distancing/ or quarantine/ or Contact Tracing/ or mass gatherings/ OR exp Telemedicine/	exp infant/ or (foetal or foetal or fetus or foetus or preterm or premature or infan* or neonat* or baby or babies or newborn* or new-born*).tw,kf. Or exp *pregnant women/ or exp *Pregnancy/ or exp *Pregnancy Complications/ or exp *Obstetrics/ or exp *Breast Feeding/ OR exp *Maternal Health Services/ or (breast-feeding education or parturition or ante natal or antenatal* or pre natal* or prenatal* or puerper* or postnatal* or postpartum or post partum or post natal* or peripartum or peri partum or prepregnancy or pre pregnancy or preconception* or pre conception* or periconception* or peri conception* or birth or breastfe* or breast fe* or lactation* or stillbirth* or miscarriage* or pregnancy or pregnancies or pregnant or perinatal or peri-natal or mother or mothers or maternal).tw,kf.	Epidemiologic studies/ or exp case control studies/ or exp cohort studies/ or Case-control.tw. or (cohort adj (study or studies)).tw. or Cohort analy$.tw. or (Follow up adj (study or studies)).tw. or (observational adj (study or studies)).tw. or cross-sectional.tw. or cross-sectional studies/ or (longterm or long-term or repeat* or serial or longitudinal* or follow-up or followup or retrospective* or prospective* or interrupted-time-series or natural-experiment*).tw. OR Randomized controlled trial.pt OR Randomi#ed.tw OR placebo.tw,dt.fs OR Randomly.tw.OR trial.tw. OR groups.tw
**EMBASE**	coronavirus disease 2019/ or SARS coronavirus/ or experimental coronavirus disease 2019/ or exp severe acute respiratory syndrome coronavirus 2/ OR (2019-novel or 2019nCoV or 2019-nCoV or COVID-19 or COVID19 or COVID-2019 or COVID2019 or CONVID-19 or CONVID19 or CORVID-19 or CORVID19 or CoV2 or CoV-2 or HCoV* or Ncov* or Ncorona* or Ncorono* or NcovChina* or NcovChinese* or NcovHubei* or NcovWuhan* or SARS2 or SARS-2 or SARScoronavirus2 or SARScoronavirus-2 or	fiscal policy/ or public policy/ or health care policy OR (Lockdown* or lock-down* or closure* or containment* or restriction* or density-limit* or cancel* or postpone* or quarantine* or isolation or stay-at-home* or close-contact* or household-contact* or redeploy* or JobKeeper* or JobSeeker* or unemploy* or subsid* or income* or tax-relief* or mortgage-relief* or rent-relief* or financial or economic* or cash-transfer* or cash-payment or allowance* or paid-sick-leave or travel* or radius or childcare* or child-care* or government-support* or welfare*	exp infant/ or (foetal or foetal or fetus or foetus or preterm or premature or infan* or neonat* or baby or babies or newborn* or new-born*).tw,kf,dq OR exp pregnancy/ or exp pregnancy disorder/ or exp obstetric procedure/ or exp breast feeding/ or exp breast feeding education/ or exp birth/ or exp childbirth/ or maternal health service/ or (breast-feeding education or parturition or ante natal or antenatal* or pre natal* or prenatal* or puerper* or postnatal* or postpartum or post partum or post natal* or peripartum or peri partum or	Clinical study/ or Case control study/ or Family study/ or Longitudinal study/ or Retrospective study/ or (Prospective study/ not Randomized controlled trials/) or Cohort analysis/ or (Cohort adj (study or studies)).mp. or (Case control adj (study or studies)).tw. or (follow up adj (study or studies)).tw. or (observational adj (study or studies)).tw. or (epidemiologic$ adj (study or studies)).tw. or (cross sectional adj (study or studies)).tw. or (longterm or long-term or repeat* or serial or longitudinal* or follow-up or followup or

Database	COVID-19	Policies	Maternal and infant	Study design
	SARScoronovirus2 or SARScoronovirus-2 or SARSCov19 or SARSCov-19 or SARS-CoV-2 or SARSCoV-2 or SARSCoV2 or WN-CoV or WNCoV or wuhan-virus).tw,kf,dq,ot. OR ((pneumonia* or outbreak* or respiratory-illness* or respiratory-disease* or respiratory-symptom* or seafood-market* or food-market* or wildlife) and (Wuhan or China or Chinese or Hubei or Huanan)).tw,kf,dq,ot. OR<br>((new or novel or nouveau or “19” or “2019” or Wuhan or Hubei or Huanan or China or Chinese) adj3 (coronavirus* or corona virus* or betacoronavirus* or CoV or HCoV)).ti,ab,kf,dq,ot.. OR (longCOVID* or postCOVID* or postcoronavirus* or postSARS*).ti,ab,kf,ot. OR (*coronavirus/ or *betacoronavirus/ or *coronavirus infections/) and (*disease outbreaks/ or *epidemics/ or *pandemics/) OR<br>((coronavirus* or corona-virus* or betacoronavirus*) adj3 (pandemic* or epidemic* or outbreak* or crisis)). tw,kf,dq,ot. OR Severe-acute-respiratory-syndrome-coronavirus-2.hw OR Coronavirus-disease-2019.hw	or social-security* or mass-gathering* or social-distanc* or physical-distanc* or mask* or visitor-restriction* or public-health-measure* or goverment-regulation*).tw,kf,dq OR (telemedicine or tele-medicine or remote-monitor* or long-distance-monitor* or remote-sensing or telesensing or tele-sensing or telemonitoring or tele-monitoring or telehealth or tele-health or teleconsultation* or tele-consultation* or virtual-care or virtual-health or mobile-health or m-health or app or apps or e-health or ehealth or telepractice or tele-practice or technology-based or emerging-technolog* or digital-health).tw,kf,dq OR<br>(policy or policies or government-order? or mandate*).tw,kf,dq.OR social distancing/ or exp quarantine/ or contact examination/ OR<br>exp telemedicine/	prepregnancy or pre pregnancy or preconception* or pre conception* or periconception* or peri conception* or birth or breastfe* or breast fe* or lactation* or stillbirth* or miscarriage* or pregnancy or pregnancies or pregnant or perinatal or peri-natal or mother or mothers or maternal).tw,kf,dq	retrospective* or prospective* or interrupted-time-series or natural-experiment*).tw. OR (Randomized controlled trial/ or randomized controlled trials/ or "randomized controlled trial (topic)"/ or Controlled clinical study/ or random$.ti,ab. or randomization/ or intermethod comparison/ or placebo.ti,ab. or (compare or compared or comparison).ti. or ((evaluated or evaluate or evaluating or assessed or assess) and (compare or compared or comparing or comparison)).ab. or (open adj label).ti,ab. or ((double or single or doubly or singly) adj (blind or blinded or blindly)).ti,ab. or single blind procedure/ or double blind procedure/ or parallel group$1.ti,ab. or (crossover or cross over).ti,ab. or ((assign$ or match or matched or allocation) adj5 (alternate or group$1 or intervention$1 or patient$1 or subject$1 or participant$1)).ti,ab. or (assigned or allocated).ti,ab. or (controlled adj7 (study or design or trial)).ti,ab. or (volunteer or volunteers).ti,ab. or human experiment/ or trial.ti.) not (((random$ adj sampl$ adj7 ("cross section$" or questionnaire$1 or survey$ or database$1)).ti,ab. not (comparative study/ or controlled study/ or randomi?ed controlled.ti,ab. or randomly assigned.ti,ab.)) or (Cross-sectional study/ not (randomized controlled trial/ or controlled clinical study/ or controlled study/ or randomi?ed controlled.ti,ab. or control group$1.ti,ab.)) or (((case adj control$) and random$) not randomi?ed controlled).ti,ab. or (Systematic review not (trial or study)).ti. or (nonrandom$ not random$).ti,ab. or "Random field$".ti,ab. or (random cluster adj3 sampl$).ti,ab. or ((review.ab. and review.pt.) not trial.ti.) or ("we searched".ab. and (review.ti. or review.pt.)) or "update review".ab. or (databases adj4 searched).ab. or ((rat or rats or mouse or mice or swine or porcine or murine or sheep or lambs or pig or pigs or piglet or piglets or rabbit or rabbits or cat or cats or dog or dogs or cattle or bovine or monkey or monkeys or trout or marmoset or marmosets).ti. and animal experiment/) or (Animal

Database	COVID-19	Policies	Maternal and infant	Study design
				experiment/ not (human experiment/ or human/)))
**PubMed**	((((“2019-novel” OR “2019nCoV” OR “2019-nCoV” OR “COVID-19” OR “COVID19” OR “COVID-2019” OR “COVID2019” OR “CONVID-19” OR “CONVID19” OR “CORVID-19” OR “CORVID19” OR “CoV2” OR “CoV-2” OR “HCoV*” OR “Ncov*” OR “Ncorona*” OR “Ncorono*” OR “NcovChina*” OR “NcovChinese*” OR “NcovHubei*” OR “NcovWuhan*” OR “SARS2” OR “SARS-2” OR “SARScoronavirus2” OR “SARScoronavirus-2” OR “SARScoronovirus2” OR “SARScoronovirus-2” OR “SARSCov19” OR “SARSCov-19” OR “SARS-CoV-2” OR “SARSCoV-2” OR “SARSCoV2” OR “WN-CoV” OR “WNCoV” OR “wuhan-virus”) OR ((“pneumonia*” OR “outbreak*” OR “respiratory-illness*” OR “respiratory-disease*” OR “respiratory-symptom*” OR “seafood-market*” OR “food-market*” OR “wildlife”) AND (“Wuhan” OR “China” OR “Chinese” OR “Hubei” OR “Huanan”)) OR ((“new” OR “novel” OR “nouveau” OR “19” OR “2019” OR “Wuhan” OR “Hubei” OR “Huanan” OR “China” OR “Chinese”) AND (“coronavirus*” OR “corona virus*” OR “betacoronavirus*” OR “CoV” OR “HCoV”)) OR (“longCOVID*” OR “postCOVID*” OR “postcoronavirus*” OR “postSARS*”) OR ((“coronavirus*” OR “corona-virus*” OR “betacoronavirus*”) AND (“pandemic*” OR “epidemic*” OR “outbreak*” OR “crisis”)))	((“Lockdown*”[Title/Abstract] OR “lock-down*”[Title/Abstract] OR “closure*”[Title/Abstract] OR “containment*”[Title/Abstract] OR “restriction*”[Title/Abstract] OR “density-limit*”[Title/Abstract] OR “cancel*”[Title/Abstract] OR “postpone*”[Title/Abstract] OR “quarantine*”[Title/Abstract] OR “isolation”[Title/Abstract] OR “stay-at-home*”[Title/Abstract] OR “close-contact*”[Title/Abstract] OR “household-contact*”[Title/Abstract] OR “redeploy*”[Title/Abstract] OR “JobKeeper*”[Title/Abstract] OR “JobSeeker*”[Title/Abstract] OR “unemploy*”[Title/Abstract] OR “subsid*”[Title/Abstract] OR “income*”[Title/Abstract] OR “tax-relief*”[Title/Abstract] OR “mortgage-relief*”[Title/Abstract] OR “rent-relief*”[Title/Abstract] OR “financial”[Title/Abstract] OR “economic*”[Title/Abstract] OR “cash-transfer*”[Title/Abstract] OR “cash-payment”[Title/Abstract] OR “allowance*”[Title/Abstract] OR “paid-sick-leave”[Title/Abstract] OR “travel*”[Title/Abstract] OR “radius”[Title/Abstract] OR “childcare*”[Title/Abstract] OR “child-care*”[Title/Abstract] OR “government-support*”[Title/Abstract] OR “welfare*”[Title/Abstract] OR “social-security*”[Title/Abstract] OR “mass-gathering*”[Title/Abstract] OR “social-distanc*”[Title/Abstract] OR “physical-distanc*”[Title/Abstract] OR “mask*”[Title/Abstract] OR “visitor-restriction*”[Title/Abstract] OR “public-health-measure*”[Title/Abstract] OR “goverment-regulation*”[Title/Abstract] OR “contact-trac*”[Title/Abstract]) OR (“telemedicine”[Title/Abstract] OR “tele-medicine”[Title/Abstract] OR “remote-monitor*”[Title/Abstract] OR “long-distance-monitor*”[Title/Abstract] OR “remote-sensing”[Title/Abstract] OR “telesensing”[Title/Abstract] OR “tele-sensing”[Title/Abstract] OR “telemonitoring”[Title/Abstract] OR “tele-monitoring”[Title/Abstract] OR “telehealth”[Title/Abstract] OR “tele-health”[Title/Abstract] OR “teleconsultation*”[Title/Abstract] OR “tele-consultation*”[Title/Abstract] OR “virtual-care”[Title/Abstract] OR “virtual-health”[Title/Abstract] OR “mobile-health”[Title/Abstract] OR “m-health”[Title/Abstract] OR “app”[Title/Abstract] OR	((“foetal”[Title/Abstract] OR “fetus”[Title/Abstract] OR “foetus”[Title/Abstract] OR “preterm”[Title/Abstract] OR “premature”[Title/Abstract] OR “infan*”[Title/Abstract] OR “neonat*”[Title/Abstract] OR “baby”[Title/Abstract] OR “babies”[Title/Abstract] OR “newborn*”[Title/Abstract] OR “new-born*”[Title/Abstract]) OR (“breast-feeding”[Title/Abstract] OR “parturition”[Title/Abstract] OR “ante natal”[Title/Abstract] OR “antenatal*”[Title/Abstract] OR “pre natal*”[Title/Abstract] OR “prenatal*”[Title/Abstract] OR “puerper*”[Title/Abstract] OR “postnatal*”[Title/Abstract] OR “postpartum”[Title/Abstract] OR “post partum”[Title/Abstract] OR “post natal*”[Title/Abstract] OR “peripartum”[Title/Abstract] OR “peri partum”[Title/Abstract] OR “prepregnancy”[Title/Abstract] OR “pre pregnancy”[Title/Abstract] OR “preconception*”[Title/Abstract] OR “pre conception*”[Title/Abstract] OR “periconception*”[Title/Abstract] OR “peri conception*”[Title/Abstract] OR “birth”[Title/Abstract] OR “breastfe*”[Title/Abstract] OR “breast fe*”[Title/Abstract] OR “lactation*”[Title/Abstract] OR “stillbirth*”[Title/Abstract] OR “miscarriage*”[Title/Abstract] OR “pregnancy”[Title/Abstract] OR “pregnancies”[Title/Abstract] OR “pregnant”[Title/Abstract] OR “perinatal”[Title/Abstract] OR “peri-natal”[Title/Abstract] OR “mother”[Title/Abstract] OR “mothers”[Title/Abstract] OR “maternal”[Title/Abstract] OR “Obstetric*”[Title/Abstract] OR “childbirth”[Title/Abstract]))	("Epidemiologic"[Title/Abstract] OR "cohort-stud*"[Title/Abstract] OR "Case-control"[Title/Abstract] OR "Cohort analy*"[Title/Abstract] OR "observational"[Title/Abstract] OR "cross-sectional"[Title/Abstract] OR "longterm"[Title/Abstract] OR "long-term"[Title/Abstract] OR "repeat*"[Title/Abstract] OR "serial"[Title/Abstract] OR "longitudinal*"[Title/Abstract] OR "follow-up"[Title/Abstract] OR "followup"[Title/Abstract] OR "retrospective*"[Title/Abstract] OR "prospective*"[Title/Abstract] OR "interrupted-time-series"[Title/Abstract] OR "natural-experiment*"[Title/Abstract]) OR ((randomized controlled trial[pt]) OR (controlled clinical trial[pt]) OR (randomized[tiab] OR randomised[tiab]) OR (placebo[tiab]) OR (drug therapy[sh]) OR (randomly[tiab]) OR (trial[tiab]) OR (groups[tiab]))

Database	COVID-19	Policies	Maternal and infant	Study design
		"apps"[Title/Abstract] OR "e-health"[Title/Abstract] OR "ehealth"[Title/Abstract] OR "telepractice"[Title/Abstract] OR "tele-practice"[Title/Abstract] OR "technology-based"[Title/Abstract] OR "emerging-technolog*"[Title/Abstract] OR "digital-health"[Title/Abstract]) OR ("policy"[Title/Abstract] OR "policies"[Title/Abstract] OR "government-order*"[Title/Abstract] OR "mandate*"[Title/Abstract] OR "reform*"[Title/Abstract]))		
**Web of Science**	(All fields) "2019-novel" OR "2019nCoV" OR "2019-nCoV" OR "COVID-19" OR "COVID19" OR "COVID-2019" OR "COVID2019" OR "CONVID-19" OR "CONVID19" OR "CORVID-19" OR "CORVID19" OR "CoV2" OR "CoV-2" OR "HCoV*" OR "Ncov*" OR "Ncorona*" OR "Ncorono*" OR "NcovChina*" OR "NcovChinese*" OR "NcovHubei*" OR "NcovWuhan*" OR "SARS2" OR "SARS-2" OR "SARScoronavirus2" OR "SARScoronavirus-2" OR "SARScoronovirus2" OR "SARScoronovirus-2" OR "SARSCov19" OR "SARSCov-19" OR "SARS-CoV-2" OR "SARSCoV-2" OR "SARSCoV2" OR "WN-CoV" OR "WNCoV" OR "wuhan-virus" OR "2019-novel" OR "2019nCoV" OR "2019-nCoV" OR "COVID-19" OR "COVID19" OR "COVID-2019" OR "COVID2019" OR "CONVID-19" OR "CONVID19" OR "CORVID-19" OR "CORVID19" OR "CoV2" OR "CoV-2" OR "HCoV*" OR "Ncov*" OR "Ncorona*" OR "Ncorono*" OR "NcovChina*" OR "NcovChinese*" OR "NcovHubei*" OR "NcovWuhan*" OR "SARS2" OR "SARS-2" OR "SARScoronavirus2" OR "SARScoronavirus-2" OR "SARScoronovirus2" OR "SARScoronovirus-2" OR "SARSCov19" OR "SARSCov-19" OR "SARS-CoV-2" OR "SARSCoV-2" OR "SARSCoV2" OR "WN-CoV" OR "WNCoV" OR "wuhan-virus" OR ("pneumonia*" OR "outbreak*" OR "respiratory-illness*" OR "respiratory-disease*" OR "respiratory-symptom*" OR "seafood-market*" OR "food-market*" OR "wildlife") AND ("Wuhan" OR "China" OR "Chinese" OR "Hubei" OR "Huanan") OR ("pneumonia*"	(Title/abstract) "Lockdown*" OR "lock-down*" OR "closure*" OR "containment*" OR "restriction*" OR "density-limit*" OR "cancel*" OR "postpone*" OR "quarantine*" OR "isolation" OR "stay-at-home*" OR "close-contact*" OR "household-contact*" OR "redeploy*" OR "JobKeeper*" OR "JobSeeker*" OR "unemploy*" OR "subsid*" OR "income*" OR "tax-relief*" OR "mortgage-relief*" OR "rent-relief*" OR "financial" OR "economic*" OR "cash-transfer*" OR "cash-payment" OR "allowance*" OR "paid-sick-leave" OR "travel*" OR "radius" OR "childcare*" OR "child-care*" OR "government-support*" OR "welfare*" OR "social-security*" OR "mass-gathering*" OR "social-distanc*" OR "physical-distanc*" OR "mask*" OR "visitor-restriction*" OR "public-health-measure*" OR "goverment-regulation*" OR "contact-trac*" OR"telemedicine" OR "tele-medicine" OR "remote-monitor*" OR "long-distance-monitor*" OR "remote-sensing" OR "telesensing" OR "tele-sensing" OR "telemonitoring" OR "tele-monitoring" OR "telehealth" OR "tele-health" OR "teleconsultation*" OR "tele-consultation*" OR "virtual-care" OR "virtual-health" OR "mobile-health" OR "m-health" OR "app" OR "apps" OR "e-health" OR "ehealth" OR "telepractice" OR "tele-practice" OR "technology-based" OR "emerging-technolog*" OR "digital-health" OR"policy" OR "policies" OR "government-order*" OR "mandate*" OR "reform*"	(Title/abstract) "foetal" OR "foetal" OR "fetus" OR "foetus" OR "preterm" OR "premature" OR "infan*" OR "neonat*" OR "baby" OR "babies" OR "newborn*" OR "new-born*" OR "breast-feeding" OR "parturition" OR "ante natal" OR "antenatal*" OR "pre natal*" OR "prenatal*" OR "puerper*" OR "postnatal*" OR "postpartum" OR "post partum" OR "post natal*" OR "peripartum" OR "peri partum" OR "prepregnancy" OR "pre pregnancy" OR "preconception*" OR "pre conception*" OR "periconception*" OR "peri conception*" OR "birth" OR "breastfe*" OR "breast fe*" OR "lactation*" OR "stillbirth*" OR "miscarriage*" OR "pregnancy" OR "pregnancies" OR "pregnant" OR "perinatal" OR "peri-natal" OR "mother" OR "mothers" OR "maternal" OR "Obstetric*" OR "childbirth	"Epidemiologic" OR "cohort-stud*" OR "Case-control" OR "Cohort analy*" OR "observational" OR "cross-sectional" OR "longterm" OR "long-term" OR "repeat*" OR "serial" OR "longitudinal*" OR "follow-up" OR "followup" OR "retrospective*" OR "prospective*" OR "interrupted-time-series" OR "natural-experiment*" OR "randomized controlled trial" OR "controlled clinical" OR "randomized" OR "randomised' OR "placebo" OR "drug therapy" OR "randomly" OR "trial" OR "groups"

Database	COVID-19	Policies	Maternal and infant	Study design
	OR “outbreak*” OR “respiratory-illness*” OR “respiratory-disease*” OR “respiratory-symptom*” OR “seafood-market*” OR “food-market*” OR “wildlife”) AND (“Wuhan” OR “China” OR “Chinese” OR “Hubei” OR “Huanan”) OR(“new” OR “novel” OR “nouveau” OR “19” OR “2019” OR “Wuhan” OR “Hubei” OR “Huanan” OR “China” OR “Chinese”) AND (“coronavirus*” OR “corona virus*” OR “betacoronavirus*” OR “CoV” OR “HCoV”) OR(“new” OR “novel” OR “nouveau” OR “19” OR “2019” OR “Wuhan” OR “Hubei” OR “Huanan” OR “China” OR “Chinese”) AND (“coronavirus*” OR “corona virus*” OR “betacoronavirus*” OR “CoV” OR “HCoV”) OR “longCOVID*” OR “postCOVID*” OR “postcoronavirus*” OR “postSARS*” OR “longCOVID*” OR “postCOVID*” OR “postcoronavirus*” OR “postSARS*” OR (“coronavirus*” OR “corona-virus*” OR “betacoronavirus*”) AND (“pandemic*” OR “epidemic*” OR “outbreak*” OR “crisis”) OR (“coronavirus*” OR “corona-virus*” OR “betacoronavirus*”) AND (“pandemic*” OR “epidemic*” OR “outbreak*” OR “crisis”)			

Appendix SB - Data extraction template

The following data were extracted:

Date of access
Study identification
Journal
Year of publication
Country
Title
First author's name and contact details
Study design
Study aim
Single site or multicentre
Period of sampling
Period of data collection
Eligibility criteria of study population
Lifecourse stage of participants (antenatal, birth, postnatal 12 months)
Method of recruitment
Sample size
Comparison group criteria
Assessment of study population baseline characteristics
COVID-19 policy exposure and how this was measured
Maternal-infant health outcome(s) and how these were measured
Confounder variables and how these were measured
The Oxford COVID-19 Government Response Tracker Stringency Index (if applicable)
Methods used to prevent and address missing data
Absolute number of participants and events
Summary estimates and measures of precision and variance
Between group estimates with measures of precision and variance
Key conclusions

Appendix SC - Decision rules for application of the Newcastle-Ottawa Scale

Criteria	Scale	Explanation
**Selection**		
Representativeness of the exposed cohort	a) Truly representative of the average in the community*<br>b) Somewhat representative of the average in the community*<br>c) Selected group of users e.g. Nurses, volunteers<br>d) No description of the derivation of the cohort	Item is assessing the representativeness of exposed individuals in the community, not the representativeness of the sample of women or infants from some general population. Therefore, covid-19 policies (e.g. Lockdowns, telehealth, visitor restrictions) that were systematically applied to a defined population was given one point on the representativeness of the exposed group.
Selection of the non-exposed cohort	a) Drawn from the same community as the exposed cohort*<br>b) Drawn from a different source*<br>c) No description of the derivation of the non-exposed cohort	
Ascertainment of the exposure	a) Secure record (e.g. Government or health policy, medical records)*<br>b) Structured interview*<br>c) Written self-report	
Demonstration that outcome of interest was not present at start of study.	a) Yes*<br>b) No	
**Comparability**		This item has a maximum of two points for comparability. According to harling, et al. (2012), articles that report no differences in baseline demographics between the exposed and comparison groups or statements that the differences are not statistically significant are not sufficient for establishing comparability.[1] rather, the exposed and comparison groups need to be matched or the confounders need to be adjusted for in the analysis for the group to be considered comparable with regard to each variable accounted for.[1] if a study just reported on a subgroup analysis, this was not classified as an adjustment for a confounder. Some studies only accounted for confounders in the analysis of some outcomes and not others. In this case, a study was still given points for comparability. The authors recognise that this is not a comprehensive adjustment for all confounders as would be in a randomised control trial. However, due to the opportunistic nature of examining the covid-19 policies and the likely 'real world' factors at-play such as limitations on available routinely data collected.
Comparability of cohorts on the basis of the design or analysis	a) Study controls for maternal age (the most important factor)*	The first point was given to studies who accounted for maternal age, as identified by the study authors as being the most important confounder. If included studies did not adjust for this confounder, then these studies could not score low risk of bias.
Comparability of cohorts on the basis of the design or analysis	a) Study controls for any additional factor*	The second point was given if a study accounted for any other confounders. This included even if the study only adjusted for seasonality.[2]
**Outcome**		
Assessment of outcome	a) Independent or blind assessment stated in the paper, or confirmation of the outcome by reference to secure records (e.g. Medical records.)*<br>b) Record linkage (e.g. Identified through icd codes on database records)*<br>c) Self-report (e.g. Identified through mental health questionnaire)<br>d) No description.	
Was follow-up long enough for outcomes to occur	a) Yes*<br>b) No	Follow-up period was determined as being long enough based on each articles aims.
Adequacy of follow-up of cohorts	a) Complete follow-up, all subjects accounted for*<br>b) Subjects lost to follow-up are unlikely to introduce bias – small number lost <20%<br>c) Follow-up rate <80% and no description of those lost<br>d) No description *or unclear*	If there was no explicit statement about missing data there was no point given for follow up from the time of exposure to the collection of data.

Results from the decision rules applied to classify the Newcastle-Ottawa Scale scores in Table 4.

Low risk: 3 or 4 stars (*) in selection domain AND 1 (has to be maternal age) or 2 stars (*) in comparability domain AND 2 or 3 stars (*) in outcome/exposure domain

Moderate risk: 2 stars (*) in selection domain AND 1 or 2 stars (*) in comparability domain AND 2 or 3 stars (*) in outcome/exposure domain

High risk: 0 or 1 star (*) in selection domain OR 0 stars (*) in comparability domain OR 0 or 1 stars (*) in outcome/exposure domain

Appendix SD - Confounders applicable to the Newcastle-Ottawa Scale

Outcomes	Additional predisposing factors to maternal age	Reference
**Maternal health**		
Perinatal depression	Low education level<br>Low socioeconomic status<br>Low social support<br>History of mental ill-health<br>Smoking and substance use<br>Multipara<br>Exposure to violence<br>Adverse life events	(3)
Perinatal anxiety	Exposure to violence<br>Low social support<br>History of mental ill-health<br>Medical comorbidities<br>Adverse life events<br>Smoking and substance use<br>Coping styles	(4)
Gestational diabetes mellitus	Raised BMI<br>Ethnicity<br>Medical condition associated with diabetes development (hypothyroidism, polycystic ovarian syndrome)<br>Multipara<br>Twin pregnancy<br>Family history of diabetes<br>Previous gestational diabetes mellitus	(5)
Hypertensive disorders of pregnancy	Raised BMI<br>Previous preeclampsia<br>Nulliparity<br>Twin pregnancy<br>Family history<br>Assisted reproductive technology<br>Medical conditions associated with pre-eclampsia (chronic hypertension, antiphospholipid antibody syndrome, pregestational diabetes)<br>Previous stillbirth or placental abruption	(6-8)
**Infant health**		
Preterm birth/low birthweight	Low or raised BMI<br>Ethnicity<br>Previous preterm<br>Medical and mental health conditions (such as infection, depression, hypertension, pregestational diabetes mellitus)<br>Smoking and substance use<br>Adverse life events<br>Twin pregnancy<br>Low socioeconomic status<br>Low education<br>Single marital status	(9)
Stillbirth	Raised BMI<br>Infant sex<br>Smoking and substance use<br>Medical conditions (pregestational diabetes mellitus, infection, hypertension)<br>Air pollution<br>Rhesus disease	(10, 11)
Breast feeding	Raised BMI<br>Medical conditions (polycystic ovarian syndrome, diabetes mellitus, hypertension, depression)<br>Smoking<br>Preterm/low birth weight<br>Caesarean section<br>Intrapartum complications (blood loss)	(12)

References

1. Hartling L, Hamm M, Milne A, Vandermeer B, Santaguida PL, Ansari M, et al. Validity and Inter-Rater Reliability Testing of Quality Assessment Instruments. Rockville, MD: Agency for Healthcare Research and Quality; 2012.
2. Darrow LA, Strickland MJ, Klein M, Waller LA, Flanders WD, Correa A, et al. Seasonality of birth and implications for temporal studies of preterm birth. *Epidemiology*. 2009;20:699-706.
3. Yang K, Wu J, Chen X. Risk factors of perinatal depression in women: a systematic review and meta-analysis. BMC Psychiatry. 2022;22:63.
4. Bayrampour H, Vinturache A, Hetherington E, Lorenzetti DL, Tough S. Risk factors for antenatal anxiety: a systematic review of the literature. J Reprod Infant Psychol. 2018;36:476–503.
5. Kampmann U, Madsen L, Skajaa G, Iversen D, Moeller N, Ovesen P. Gestational diabetes: a clinical update. World J Diabetes. 2015;6:1065–72.
6. Emily B, Karyn EM, Alison LP, Joel GR. Clinical risk factors for pre-eclampsia determined in early pregnancy: systematic review and meta-analysis of large cohort studies. Bmj. 2016;353:i1753.
7. Duckitt K, Harrington D. Risk factors for pre-eclampsia at antenatal booking: systematic review of controlled studies. Bmj. 2005;330:565.
8. Chappell LC, Cluver CA, Kingdom J, Tong S. Pre-eclampsia. Lancet. 2021;398:341–54.
9. Goldenberg RL, Culhane JF, Iams JD. Epidemiology and causes of preterm birth. Lancet. 2008;371:75–84.
10. Flenady V, Koopmans L, Middleton P, Frøen JF, Smith GC, Gibbons K, et al. Major risk factors for stillbirth in high-income countries: a systematic review and meta-analysis. Lancet. 2011;377:1331–40.
11. Lawn JE, Blencowe H, Waiswa P, Amouzou A, Mathers C, Hogan D, et al. Stillbirths: rates, risk factors, and acceleration towards 2030. Lancet. 2016;387:587–603.
12. Meek JY, Noble L. Policy statement: breastfeeding and the use of human milk. Pediatrics. 2022;150:e2022057988.

Appendix SE - OxCGRT Stringency Index scores across 14 countries

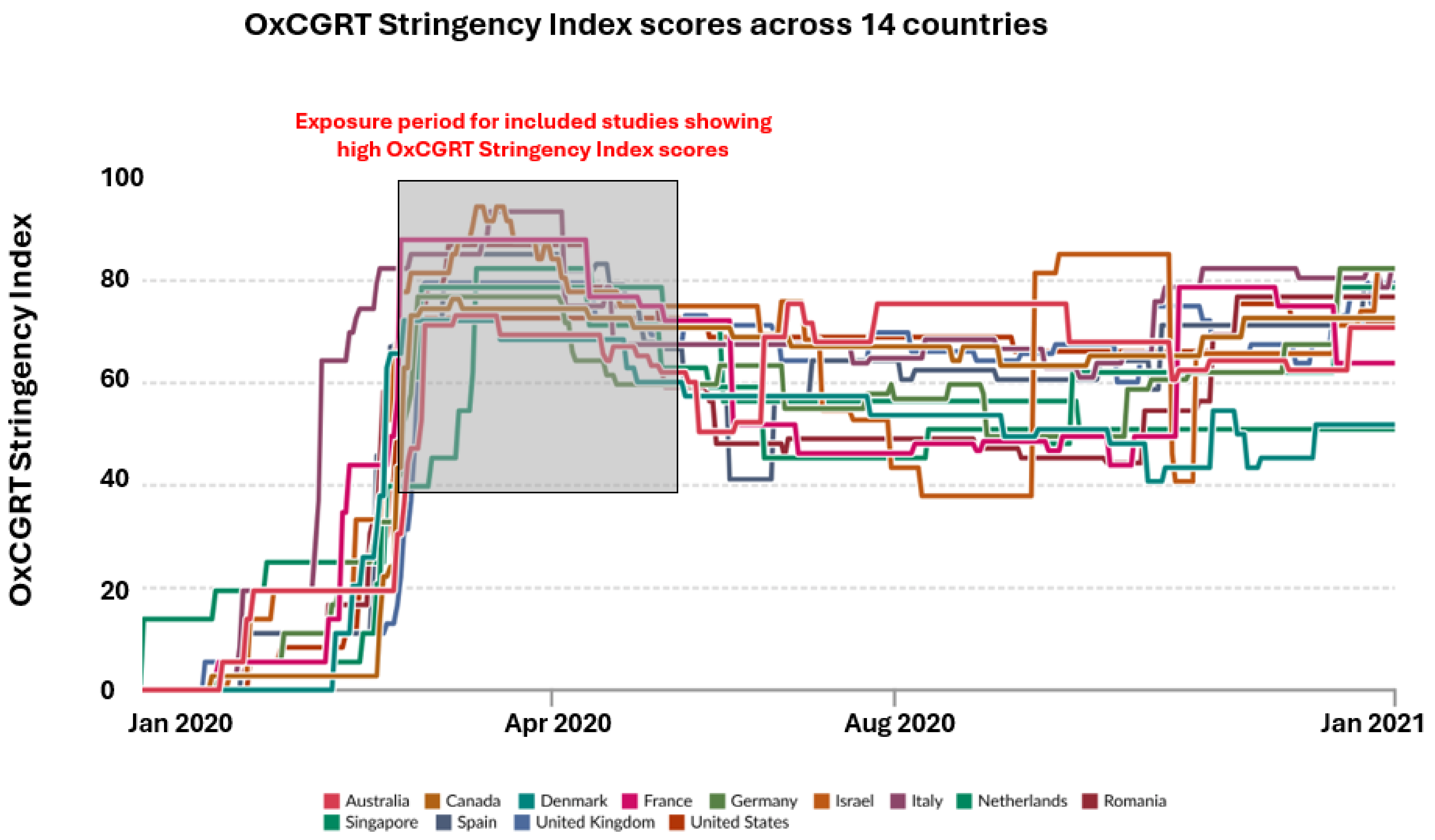

Appendix SF - Maternal and infant health outcomes

Maternal Health Outcomes

STUDY	POLICY*	OUTCOME	OR	MD	(95% CI)	lnOR[d]	ES[d]	(95% CI) [d]	RISK OF BIAS
**ANTENATAL MENTAL HEALTH**									
Morris (2021)	Lockdown	Depression symptoms		5.04[d]	(3.11, 6.97)		0.56	(0.34, 0.78)	High
Vacaru (2021)	Lockdown	Depression symptoms		0.96[d]	(0.60, 1.32)		0.22	(0.14, 0.30)	High
Silverman (2020a)	Lockdown	Depression symptoms		-1.10[d]	(-2.00, -0.20)		-0.27	(-0.50, -0.05)	High
Vacaru (2021)	Lockdown	Depression[1]		[nk1]					High
Morris (2021)	Lockdown	Anxiety symptoms		18.00[d]	(15.42, 20.58)		1.50	(1.27, 1.73)	High
Vacaru (2021)	Lockdown	Anxiety symptoms		6.70[d]	(5.97, 7.43)		0.73	(0.65, 0.81)	High
Vacaru (2021)	Lockdown	Anxiety[2]		[nk1]					High
Morris (2021)	Lockdown	Stress symptoms		5.26[d]	(4.10, 6.42)		0.97	(0.75, 1.19)	High
**POSTNATAL MENTAL HEALTH**									
Zanardo (2021)	Lockdown	Depression symptoms		1.45[d]	(0.43, 2.47)		0.32	(0.09, 0.55)	High
Zanardo (2020)	Lockdown	Depression symptoms		2.16[d]	(0.93, 3.39)		0.50	(0.21, 0.78)	High
Silverman (2020b)	Lockdown	Depression symptoms		-1.46[d]	(-2.21, -0.71)		-0.34	(-0.51, -0.16)	High
Zanardo (2021)	Lockdown	Depression[3]	2.29[d]		(1.22, 4.27)	0.83		(0.20, 1.45)	High
Zanardo (2020)	Lockdown	Depression[3]	2.97[d]		(1.41, 6.24)	1.09		(0.34, 1.83)	High
Pariente (2020)	Lockdown	Depression[1]	0.30		(0.15, 0.74)	-1.20		(-1.90, -0.30)	High
Matei (2021)	Lockdown	Depression[4]	0.33[d]		(0.00, 2.07)	-1.10		([nk2], 0.73)	High
Silverman (2020b)	Lockdown	Depression[5]	0.87[d]		(0.44, 1.72)	-0.13		(-0.81, 0.54)	High
**GESTATIONAL DIABETES**									
Harvey (2021)	Lockdown	Gestational diabetes	1.34[d]		(1.23, 1.47)	0.29		(0.20, 0.39)	Low
Garabedian (2021)	Lockdown	Gestational diabetes with insulin	0.80[d]		(0.61, 1.04)	-0.23		(-0.49, 0.04)	Low
Alshaikh (2021)	Lockdown	Gestational diabetes	1.10[rr]		(1.00, 1.21)	0.10		(0.00, 0.19)	Moderate
Ornaghi (2021)	Lockdown	Gestational diabetes	1.37[d]		(0.92, 2.04)	0.32		(-0.08, 0.72)	Moderate
Duryea (2021)	Telehealth	Gestational diabetes	1.06[d]		(0.92, 1.21)	0.05		(-0.08, 0.19)	Moderate
Boguslawski (2022)	Telehealth	Gestational diabetes	0.96[d]		(0.67, 1.37)	-0.04		(-0.40, 0.32)	Moderate
Palmer (2021)	Telehealth	Gestational diabetes low-risk group	0.99[d]		(0.88, 1.12)	-0.01		(-0.13, 0.11)	High
Palmer (2021)	Telehealth	Gestational diabetes high-risk group	1.21[d]		(0.99, 1.47)	0.19		(-0.01, 0.39)	High
**HYPERTENSIVE DISORDERS OF PREGNANCY**									
Harvey (2021)	Lockdown	Gestational hypertension	1.20[d]		(1.10, 1.31)	0.18		(0.09, 0.27)	Low
Garabedian (2021)	Lockdown	Hypertensive disorders	1.05[d]		(0.87, 1.28)	0.05		(-0.14, 0.24)	Low
Alshaikh (2021)	Lockdown	Gestational hypertension	1.24[rr]		(1.10, 1.40)	0.22		(0.10, 0.34)	Moderate
Ornaghi (2021)	Lockdown	Hypertensive disorders	1.37[d]		(0.70, 2.67)	0.31		(-0.35, 0.98)	Moderate
Duryea (2021)	Telehealth	Gestational hypertension	0.93[rr]		(0.86, 0.99)	-0.07		(-0.15, -0.01)	Moderate
Boguslawski (2022)	Telehealth	Hypertensive disorders	1.18[d]		(0.96, 1.44)	0.16		(-0.04, 0.37)	Moderate
Duryea (2021)	Telehealth	Pre-eclampsia with severe features	0.99[rr]		(0.89, 1.09)	-0.01		(-0.12, 0.09)	Moderate
Palmer (2021)	Telehealth	Pre-eclampsia low-risk	0.94[d]		(0.70, 1.27)	-0.06		(-0.36, 0.24)	High
Palmer (2021)	Telehealth	Pre-eclampsia high-risk	1.26[d]		(0.92, 1.74)	0.23		(-0.08, 0.55)	High
**HEALTH SERVICE ACCESS**									
Boguslawski (2022)	Telehealth	Gestational age at first visit		-1.00[d]	(-1.81, -0.19)		-0.12	(-0.21, -0.02)	Moderate
Duryea (2021)	Telehealth	Gestational age at first visit		[nk3]					Moderate

Duryea (2021)	Telehealth	Number of visits		0.40[d]	(0.27, 0.53)		0.11	(0.08, 0.15)	Moderate
Boguslawski (2022)	Telehealth	Number of visits		-0.20[d]	(-0.53, 0.13)		-0.06	(-0.15, 0.04)	Moderate
Soffer (2022)	Telehealth	Number of visits		[nk4]					Moderate
Duryea (2021)	Telehealth	Number of visits >=1	1.07[d]		(0.85, 1.34)		0.06	(-0.16, 0.29)	Moderate
Boguslawski (2022)	Telehealth	Number of visits initiated at <14 wks	0.75[d, r]		(0.61, 0.92)		-0.29	(-0.09, -0.48)	Moderate

[OR] Odds ratio
[MD] Means difference
[lnOR] Log odds (natural log)
[ES] Effect size
[CI] Confidence interval
[1] Edinburgh Postnatal Depression Scale (EPDS) >=13
[2] State-Trait Anxiety Inventory (STAI)=>40
[3] Edinburgh Postnatal Depression Scale (EPDS) >12
[4] Edinburgh Postnatal Depression Scale (EPDS) >13
[5] Edinburgh Postnatal Depression Scale (EPDS) =>12
[d] derived
[rr] risk ratio
[r] reversed
[nk1] missing effect size as inability to calculate from percentages provided
[nk2] missing 95% CI as uninterpretable due to small sample size
[nk3] missing mean difference as inability to calculate from median provided
[nk4] missing odds ratio as inability to calculate from absolute numbers provided
[*] specific lockdown restrictions vary and include school closures and bans on gatherings

Infant Health Outcomes

STUDY	POLICY	OUTCOME	OR	(95% CI)	lnOR	(95% CI)	RISK OF BIAS
**PRETERM BIRTHS**							
**<37 wks**							
Fresson (2022)	Lockdown	<37 wks	0.94[*]	(0.90, 0.98)	-0.06	(-0.11, -0.02)	Low
Harvey (2021)	Lockdown	<37 wks	0.86	(0.79, 0.93)	-0.15	(-0.24, -0.07)	Low
Jasper (2022)	Lockdown	<37 wks	0.96[d]	(0.88, 1.04)	-0.05	(-0.13, 0.04)	Low
Jasper (2022)	Lockdown	<37 wks spontaneous	0.60[d]	(0.34, 1.06)	-0.50	(-1.07, 0.06)	Low
Jasper (2022)	Lockdown	<37 wks iatrogenic	0.56[d]	(0.29, 1.08)	-0.59	(-1.25, 0.08)	Low
Garabedian (2021)	Lockdown	<37 wks	1.00	(0.86, 1.16)	0.00	(-0.15, 0.15)	Low
Leibovitch (2021)	Lockdown	<37 wks singleton	0.91	(0.85, 0.97)	-0.10	(-0.16, -0.03)	Moderate
Arnaez (2021)	Lockdown	<37 wks	0.97	(0.77, 1.22)	-0.03	(-0.26, 0.20)	Moderate
Arnaez (2021)	Lockdown	<37 wks multips	2.88[d]	(1.50, 5.54)	1.06	(0.40, 1.71)	Moderate
Alshaikh (2021)	Lockdown	<37 wks	0.91[rr]	(0.82, 1.01)	-0.09	(-0.20, 0.01)	Moderate
Ornaghi (2021)	Lockdown	<37 wks spontaneous	0.98[d]	(0.52, 1.84)	-0.02	(-0.66, 0.61)	Moderate
Duryea (2021)	Telehealth	<37 wks	0.96[rr]	(0.86, 1.06)	-0.04	(-0.15, 0.06)	Moderate
Boguslawski (2022)	Telehealth	<37 wks	1.06[d]	(0.81, 1.38)	0.06	(-0.21, 0.32)	Moderate
Palmer (2021)	Telehealth	<37 wks low risk	0.82[d]	(0.65, 1.03)	-0.20	(-0.43, 0.03)	High
Palmer (2021)	Telehealth	<37 wks high risk	1.10[d]	(0.91, 1.33)	0.09	(-0.10, 0.29)	High
**32-36+6 wks**							
Harvey (2021)	Lockdown	32-36+6 wks	0.89[d]	0.82, 0.97)	-0.12	(-0.20, -0.03)	Low
Jasper (2022)	Lockdown	32-36+6 wks	0.92[d]	0.84, 1.01)	-0.09	(-0.18, 0.01)	Low
Garabedian (2021)	Lockdown	32-36+6 wks	1.03[d]	0.86, 1.28)	0.03	(-0.18, 0.25)	Low
Fresson (2022)	Lockdown	35-36+6 wks	0.92[*]	0.87, 0.97)	-0.08	(-0.14, -0.03)	Low
Fresson (2022)	Lockdown	32-34+6 wks	0.95[*]	0.89, 1.01)	-0.05	(-0.12, 0.01)	Low
Arnaez (2021)	Lockdown	32-36+6 wks	0.97[d]	0.76, 1.24)	-0.03	(-0.28, 0.21)	Moderate
Hedley (2022)	Lockdown	32-36+6 wks singleton	0.96	(0.78, 1.17)	-0.04	(-0.25, 0.16)	Moderate
Leibovitch (2021)	Lockdown	32-36+6 wks singleton	0.93	(0.87, 1.00)	-0.07	(-0.14,0.00)	Moderate
Alshaikh (2021)	Lockdown	32-36+6 wks	0.94[d]	(.83, 1.07)	-0.06	(-0.18, 0.06)	Moderate
Boguslawski (2022)	Telehealth	32-36+6 wks	1.08[d]	(0.80, 1.47)	0.08	(-0.23, 0.38	Moderate
**<34 wks**							
Duryea (2021)	Telehealth	<34 wks	1.09[rr]	(0.90, 1.31)	0.09	(-0.11, 0.27)	Moderate
**<32 wks**							
Garabedian (2021)	Lockdown	<32 wks	0.90	(0.69, 1.19)	-0.11	(-0.37, 0.17)	Low
Harvey (2021)	Lockdown	<32 wks	0.90[d]	(0.76, 1.08)	-0.10	(-0.28, 0.08)	Low
Leibovitch (2021)	Lockdown	<32 wks singleton	0.75[d]	(0.61, 0.92)	-0.29	(-0.49, -0.09)	Moderate
Alshaikh (2021)	Lockdown	<32 wks	0.71[rr]	(0.52, 0.95)	-0.34	(-0.65, -0.05)	Moderate
Klumper (2021)	Lockdown	<32 wks singleton	0.94	(0.79, 1.12)	-0.06	(-0.24, 0.11)	Moderate
Klumper (2021)	Lockdown	<32 wks singleton iatrogenic	0.71	(0.53, 0.95)	-0.34	(-0.63, -0.05)	Moderate
Klumper (2021)	Lockdown	<32 wks singleton spontaneous	1.13	(0.91, 1.40)	0.12	(-0.09, 0.34)	Moderate
Klumper (2021)	Lockdown	<32 wks multips	1.20	(0.82, 1.75)	0.18	(-0.20, 0.56)	Moderate
Klumper (2021)	Lockdown	<32 wks multips iatrogenic	0.41	(0.15, 1.16)	-0.89	(-1.90, 0.15)	Moderate
Klumper (2021)	Lockdown	<32 wks multips spontaneous	1.55	(1.03, 2.36)	0.44	(0.03, 0.86)	Moderate
Boguslawski (2022)	Telehealth	<32 wks	0.99[d]	(0.61, 1.60)	-0.01	(-0.50, 0.47)	Moderate

STUDY	POLICY	OUTCOME	OR	(95% CI)	lnOR	(95% CI)	RISK OF BIAS
**28-31+6 wks**							
Jasper (2022)	Lockdown	28-31+6 wks	1.00[d]	(0.76, 1.31)	0.00	(-0.27, 0.27)	Low
Fresson (2022)	Lockdown	28-31+6 wks	1.01[*]	(0.93, 1.10)	0.01	(-0.07, 0.10)	Low
Alshaikh (2021)	Lockdown	28-31+6 wks	0.71[d]	(0.48, 1.03)	-0.35	(-0.73, 0.03)	Moderate
Klumper (2021)	Lockdown	28-31+6 wks singleton	0.91[d]	(0.74, 1.13)	-0.09	(-0.31, 0.12)	Moderate
Klumper (2021)	Lockdown	28-31+6 wks singleton iatrogenic	0.78[d]	(0.57, 1.09)	-0.24	(-0.57, 0.08)	Moderate
Klumper (2021)	Lockdown	28-31+6 wks singleton spontaneous	1.03[d]	(0.77, 1.36)	0.03	(-0.26, 0.31)	Moderate
Klumper (2021)	Lockdown	28-31+6 wks multips	0.81[d]	(0.50, 1.31)	-0.22	(-0.70, 0.27)	Moderate
Klumper (2021)	Lockdown	28-31+6 wks multips iatrogenic	0.38[d]	(0.12, 1.17)	-0.97	(-2.10, 0.16)	Moderate
Klumper (2021)	Lockdown	28-31+6 wks multips spontaneous	1.01[d]	(0.60, 1.75)	0.02	(-0.52, 0.56)	Moderate
Arnaez (2021)	Lockdown	28+31+6 wks	0.81[d]	(0.35, 1.85)	-0.22	(-1.05, 0.62)	Moderate
Hedley (2022)	Lockdown	28-31+6 wks	0.80	(0.41, 1.56)	-0.22	(-0.89, 0.44)	Moderate
Cesano (2021)	Lockdown	>=28-<34 wks	1.36[d]	(0.70, 2.61)	0.30	(-0.35, 0.96)	Moderate
**<28 wks**							
Jasper (2022)	Lockdown	20-27+6 wks	1.21[d]	(0.96, 1.53)	0.19	(-0.04, 0.42)	Low
Fresson (2022)	Lockdown	<28 wks	0.98[*]	(0.82, 1.17)	-0.02	(-0.20, 0.16)	Low
Cesano (2021)	Lockdown	<28 wks	2.42[d]	(0.54, [nk4])	0.88	(-0.62, [nk4])	Moderate
Alshaikh (2021)	Lockdown	<28 wks	0.70[d]	(0.43, 1.15)	-0.36	(-0.84, 0.14)	Moderate
Klumper (2021)	Lockdown	<28 wks singleton	1.01	(0.75, 1.34)	0.01	(-0.29, 0.29)	Moderate
Klumper (2021)	Lockdown	<28 wks singleton iatrogenic	0.53	(0.29, 0.97)	-0.63	(-1.24, -0.03)	Moderate
Klumper (2021)	Lockdown	<28 wks singleton spontaneous	1.31	(0.94, 1.83)	0.27	(-0.06, 0.60)	Moderate
Klumper (2021)	Lockdown	<28 wks multips	2.43	(1.35, 4.39)	0.89	(0.30, 1.48)	Moderate
Klumper (2021)	Lockdown	<28 wks multips iatrogenic	0.58	(0.07, 4.74)	-0.54	(-2.66, 1.56)	Moderate
Klumper (2021)	Lockdown	<28 wks multips spontaneous	2.97	(1.58, 5.58)	1.09	(0.46, 1.72)	Moderate
Hedley (2022)	Lockdown	<=27+6 wks singleton	0.27	(0.07, 0.86)	-1.31	(-2.66, -0.15)	Moderate
Morgan (2022)	Lockdown	<27 wks (lockdown 1)	0.88[rar**]	(0.60, 1.28)	-0.13	(-0.51, 0.25)	Moderate
Morgan (2022)	Lockdown	<27 wks (lockdown 2)	0.81[rar**]	(0.51, 1.28)	-0.21	(-0.67, 0.25)	Moderate
Arnaez (2021)	Lockdown	23-27+6 wks	1.60[d]	(0.74, 3.44)	0.47	(-0.30, 1.23)	Moderate
**BIRTHWEIGHT**							
Harvey (2021)	Lockdown	<2500g	0.87[d]	(0.80, 0.95)	-0.14	(-0.23, -0.05)	Low
Garabedian (2021)	Lockdown	<2500g	0.97	(0.84, 1.13)	-0.03	(-0.17, 0.12)	Low
Harvey (2021)	Lockdown	<1500g	0.92[d]	(0.76, 1.10)	-0.09	(-0.28, 0.10)	Low
Garabedian (2021)	Lockdown	<1500g	0.84	(0.64, 1.10)	-0.17	(-0.45, 0.10)	Low
Alshaikh (2021)	Lockdown	<1500g	0.62[rr]	(0.45, 0.86)	-0.48	(-0.80, -0.15)	Moderate
Arnaez (2021)	Lockdown	<1500g	1.49[d]	(0.88, 2.54)	0.40	(-0.13, 0.93)	Moderate
Alshaikh (2021)	Lockdown	<1000g	0.44[rr]	(0.23, 0.84)	-0.82	(-1.47, -0.17)	Moderate
Arnaez (2021)	Lockdown	<1000g	2.32[d]	(1.13, 4.77)	0.84	(0.12, 1.56)	Moderate
Arnaez (2021)	Lockdown	<1000g singleton	1.19	(0.44, 3.23)	0.17	(-0.82, 1.17)	Moderate
Cesano (2021)	Lockdown	unknown SGA	3.31[d]	(1.26, 8.67)	1.20	(0.23, 2.16)	Moderate
Soffer (2022)	Telehealth	SGA	0.69[d]	(0.52, 0.93)	-0.36	(-0.66, -0.07)	Moderate
Palmer (2021)	Telehealth	SGA singleton low risk	0.97[d]	(0.82, 1.14)	-0.03	(-0.20, 0.14)	High
Palmer (2021)	Telehealth	SGA singleton high risk	0.92[d]	(0.69, 1.22)	-0.09	(-0.37, 0.20)	High
**PERINATAL MORTALITY**							
Quibel (2022)	Lockdown	Neonatal mortality	0.86	(0.37, 1.82)	-0.15	(-0.99, 0.60)	Low
Hedley (2022)	Lockdown	Perinatal mortality singleton	0.86	(0.44, 1.66)	-0.15	(-0.82, 0.51)	Moderate

STUDY	POLICY	OUTCOME	OR	(95% CI)	lnOR	(95% CI)	RISK OF BIAS
Hedley (2022)	Lockdown	Perinatal mortality <=7 days singleton	0.65	(0.17, 2.26)	-0.43	(-1.77, 0.82)	Moderate
Hedley (2022)	Lockdown	Perinatal mortality <24 hours singleton	0.52	(0.08, 2.44)	-0.65	(-2.53, 0.89)	Moderate
Speyer (2021)	Lockdown	Neonatal mortality	1.20[d]	(0.76, 1.88)	0.18	(-0.27, 0.63)	Moderate
**STILLBIRTH**							
Garabedian (2021)	Lockdown	Stillbirth	0.81	(0.39, 1.37)	-0.21	(-0.94, 0.31)	Low
Jasper (2022)	Lockdown	Stillbirth >=20 wks	5.44[d]	(1.03, 28.67)	1.69	(0.03, 3.36)	Low
Fresson (2022)	Lockdown	Stillbirth	1.05[*]	(0.96, 1.15)	0.05	(-0.04, 0.14)	Low
Fresson (2022)	Lockdown	Stillbirth spontaneous <37 wks	0.98[*]	(0.88, 1.11)	-0.02	(-0.13, 0.10)	Low
Fresson (2022)	Lockdown	Stillbirth spontaneous >=37 wks	1.07[*]	(0.88, 1.31)	0.07	(-0.13, 0.27)	Low
Alshaikh (2021)	Lockdown	Stillbirth	0.90[rr]	(0.58, 1.39)	-0.11	(-0.54, 0.33)	Moderate
Alshaikh (2021)	Lockdown	Stillbirth excluding terminations	0.89[rr]	(0.52, 1.50)	-0.12	(-0.65, 0.41)	Moderate
Hedley (2022)	Lockdown	Stillbirth	0.97	(0.43, 2.17)	-0.03	(-0.84, 0.77)	Moderate
Hedley (2022)	Lockdown	Stillbirth <=27+6 wks	0.55	(0.08, 3.28)	-0.60	(-2.53, 1.19)	Moderate
Hedley (2022)	Lockdown	Stillbirth 28-31+6 wks	1.57	(0.21, 13.43)	0.45	(-1.56, 2.60)	Moderate
Hedley (2022)	Lockdown	Stillbirth 32-36+6 wks	1.00	(0.06, 15.94)	0.00	(-2.81, 2.77)	Moderate
Speyer (2021)	Lockdown	Stillbirth	1.02[d]	(0.43, 2.44)	0.02	(-0.85, 0.89)	Moderate
Arnaez (2021)	Lockdown	Stillbirth	1.22	(0.45, 3.23)	0.20	(-0.80, 1.17)	Moderate
Cesano (2021)	Lockdown	Stillbirth	[nk5]				Moderate
Ornaghi (2021)	Lockdown	Stillbirth	1.14[d]	[nk6]	0.13[d]	[nk6]	Moderate
Morgan (2022)	Lockdown	Stillbirth (lockdown 1)	1.08[rar**]	(0.78, 1.51)	0.07	(-0.25, 0.14)	Moderate
Morgan (2022)	Lockdown	Stillbirth (lockdown 2)	1.29[rar**]	(0.87, 1.93)	0.25	(-0.14, 0.66)	Moderate
Duryea (2021)	Telehealth	Stillbirth	0.80[rr]	(0.50, 1.29)	-0.22	(-0.69, 0.25)	Moderate
Palmer (2021)	Telehealth	Stillbirth low risk	0.92[d***]	(0.50, 1.70)	-0.08	(-0.70, 0.53)	High
Palmer (2021)	Telehealth	Stillbirth high risk	1.12[d****]	(0.63, 2.00)	0.12	(-0.46, 0.69)	High
**INFANT NUTRITION**							
**Breast feeding**							
Cesano (2021)	Lockdown	Breast feeding within 2 hours	1.81[d]	(1.51, 2.17)	0.59	(0.41, 0.78)	Moderate
Speyer (2021)	Lockdown	Exclusive breast feeding	0.99[d]	(0.92, 1.05)	-0.01	(-0.08, 0.05)	Moderate
Boguslawski (2022)	Telehealth	Exclusive breast feeding	[nk7]				Moderate
Arias (2022)	Telehealth	Breast feeding at postpartum visit	0.90[t]	(0.68, 1.18)	-0.11	(-0.39, 0.17)	Moderate
Zanardo (2021)	Lockdown	Exclusive breast feeding at discharge	0.46[d]	(0.27, 0.81)	-0.77	(-1.32, -0.21)	High
Latorre (2021)	Lockdown	Exclusive breast feeding at discharge	[nk8]				High
Latorre (2021)	Lockdown	Exclusive breast feeding at discharge-day 30 postpartum	[nk8]				High
Latorre (2021)	Lockdown	Exclusive breast feeding at day 30 postpartum	[nk8]				High
Latorre (2021)	Lockdown	Exclusive breast feeding at day 90 postpartum	[nk8]				High
Latorre (2021)	Lockdown	Exclusive breast feeding at day 90 postpartum	[nk8]				High
**Formula/mixed feeding**							
Speyer (2021)	Lockdown	Formula-feeding at discharge	0.97[d]	(0.91, 1.03)	-0.03	(-0.09, 0.03)	Moderate
Speyer (2021)	Lockdown	Mixed feeding at discharge	1.15[d]	(1.05, 1.26)	0.14	(0.05, 0.23)	Moderate
Zanardo (2021)	Lockdown	Complementary feeding at discharge	2.56[d]	(1.40, 4.69)	0.94	(0.33, 1.54)	High
Zanardo (2021)	Lockdown	Formula feeding at discharge	1.22[d]	(0.35, 4.27)	0.20	(-1.06, 1.45)	High

[OR] Odds ratio
[lnOR] Log odds
[CI] Confidence interval
[d] derived

[rr] risk ratio
[rar] rate ratio
[*] reference group observed preterm vs expected preterm
[**] reference group exposure period vs previous five years
[***] reference group all low-risk women
[****] reference group all high-risk women
[t] error in the study incorrectly presented as 0.09 instead of 0.9
[nk5] missing odds ratio and/or 95% CI as uninterpretable due to small sample size
[nk6] missing 95% CI as uninterpretable due to small sample size
[nk7] missing odds ratios due to inability to calculate with there being missing participants from unspecified denominators
[nk8] missing odds ratio due to inability to calculate from result provided as percentage change

Infant Emergency Department (ED) Admissions

STUDY	POLICY	OUTCOME	OR	(95% CI)	lnOR	(95% CI)	RISK OF BIAS
**Paediatric ED admissions**							
Chaiyachati (2020)	Lockdown	<1-yr olds vs all <=21-yr-olds	1.30[d]	(1.18, 1.44)	0.26	(0.16, 0.36)	Moderate
Silvagni (2021)	Lockdown	<1-yr olds vs all <=14-yr-olds	1.38[d]	(1.17, 1.62)	0.32	(0.15, 0.48)	Moderate
Silvagni (2021)	Lockdown	>28 days-<1-year olds vs all <=14-yr-olds	1.17[d]	(0.98, 1.40)	0.15	(-0.02, 0.33)	Moderate
Silvagni (2021)	Lockdown	<28 days vs all <=14-yr-olds	2.38[d]	(1.69, 3.36)	0.87	(0.52, 1.21)	Moderate
Soo (2021)	Lockdown	<1-yr olds vs all <=18-yr-olds	3.02[d]	(1.24, 7.34)	1.10	(0.22, 1.99)	Moderate
Dopfer (2020)	Lockdown	<1-yr olds vs all paediatric age groups	[nk9]				Moderate
Bothara (2021)	Lockdown	<1-yr olds vs all <16-yr-olds	1.47[d]	(1.16, 1.87)	0.39	(0.15, 0.63)	High
Bothara (2021)	Lockdown	6-12 months vs all <16-yr-olds	[nk8]				High
Bothara (2021)	Lockdown	3-6 months vs all <16-yr-olds	[nk8]				High
Bothara (2021)	Lockdown	<3 months vs all <16-yr-olds	[nk8]				High
Lucero (2020)	Lockdown	<1-year olds vs all <18-yr-olds	1.00[d]	(0.96, 1.04)	0.00	(-0.04, 0.04)	High

STUDY	POLICY	OUTCOME	PERCENTAGE CHANGE	RISK OF BIAS
**Paediatric ED admissions**				
Keays (2020)	Lockdown	ED admission <1-year olds	-30 [d]	Moderate
Soo (2021)	Lockdown	ED admission <=1-year olds	-27 [d]	Moderate
Chaiyachati (2020)	Lockdown	ED admission <1-year olds	-58 [d]	Moderate
Silvagni (2021)	Lockdown	ED admission <1-year olds	-58 [d]	Moderate
Silvagni (2021)	Lockdown	ED admission 28 days-1-year olds	-63 [d]	Moderate
Silvagni (2021)	Lockdown	ED admission <28 days	-24 [d]	Moderate
Bothara (2021)	Lockdown	ED admission <1-year olds	-35 [d]	Moderate
Bothara (2021)	Lockdown	ED admission 6–12 months	-33	Moderate
Bothara (2021)	Lockdown	ED admission 3-6 months	-26	Moderate
Bothara (2021)	Lockdown	ED admission <3 months	-40	Moderate

[d] derived
[nk8] missing odds ratio due to inability to calculate from result provided as percentage change
[nk9] missing odds ratio due to inability to calculate from result provided as daily mean
